# From onset to healing: temporal dynamics of microbial communities pinpoint *Midichloria*-like organism’s key role in the development of the fish skin disease red mark syndrome

**DOI:** 10.1186/s42523-026-00588-z

**Published:** 2026-08-19

**Authors:** Giulia Zarantonello, Fernando Puente-Sánchez, Jacob Günther Schmidt, Argelia Cuenca

**Affiliations:** 1 Section for Fish and Shellfish Diseases, National Institute for Aquatic Resources (DTU Aqua), Technical University of Denmark (DTU), Kongens Lyngby, Denmark; 2 Division of Microbial Ecology, Department of Aquatic Sciences and Assessment, Swedish University of Agricultural Sciences (SLU), Uppsala, Sweden

**Keywords:** Strawberry disease (SD), Red mark syndrome (RMS), *Midichloria*-like organism (MLO), Skin microbiome, Aquaculture, eDNA, Teleost, “*Ca.* Branchiomonas”

## Abstract

**Background:**

Red mark syndrome (RMS) is an infectious disease affecting rainbow trout (*Oncorhynchus mykiss*), especially at market size, forcing farmers to downgrade the product with heavy economic repercussions. The causative agent of RMS has not been established according to Koch’s postulates, since possible candidates have not been isolated and propagated in vitro. While the 16S rRNA gene of a *Midichloria*-like organism (MLO) is consistently detected in active skin lesions, the role of other bacteria in the disease has not been excluded. In this work, we provide a temporal perspective to elucidate the relationships between the bacteriome in rainbow trout skin and water, during the development and resolution of clinical disease in naive fish infected by cohabitation with RMS-affected fish in the same tank.

**Results:**

We quantified the MLO by qPCR in skin and, for the first time, in water using environmental DNA, showing that its quantity in both sample types corresponds with disease progression. Using 16S rRNA gene profiling, we provide further evidence that the MLO is likely the primary pathogen triggering RMS, as it was the only significantly enriched taxon in active lesions. Furthermore, the skin microbiome of affected fish reverted to a control-like state during healing. RMS also caused changes in the overall skin microbiome at peak pathology: the relative abundance of “*Ca*. Branchiomonas” and an unclassified gammaproteobacterium increased in apparently healthy skin areas of RMS-affected fish while remaining low in controls, suggesting they may be opportunistic bacteria implicated in skin dysbiosis.

**Conclusions:**

Our findings on the temporal dynamics of the skin microbiome during disease progression and recovery strengthen the evidence that *Midichloria*-like organism is the primary pathogen of RMS. The observed shifts in other bacterial taxa suggest possible secondary roles in disease-associated skin dysbiosis. Finally, detection of MLO in environmental DNA from water provides new insights into RMS transmission and potential monitoring strategies.

**Supplementary Information:**

The online version contains supplementary material available at 10.1186/s42523-026-00588-z.

## Introduction

Red mark syndrome (RMS), also known as strawberry disease (SD) or cold water SD (CWSD), is a skin condition affecting rainbow trout (*Oncorhynchus mykiss*) and reported in freshwater aquaculture. RMS is characterized by multifocal, bright red inflammatory skin lesions, typically raised at the lesion site, and it predominantly affects fish at market size. The disease is generally not lethal, does not seem to cause changes in growth performance or behaviour in the affected individuals, and typically undergoes spontaneous resolution over time. However, RMS can have a morbidity up to 10–60% of the farmed population [1], and does modify the fish appearance, which causes major economic losses for fish farmers due to the reduced marketability and product downgrading.

RMS was first reported in the USA in the 1950s and 1960s [2, 3], then in Europe in the 1980s [4]. More recent reports, first from the UK in 2003 [5], and subsequently from freshwater farms all over Europe [6], as well as Chile [7], Peru [8], and suspect cases in South Korea [9], confirmed that RMS is widespread across continents. As RMS is not a listed disease [10], there is no legal requirement to report cases and prevalence data is expected to be incomplete.

The disease is of infectious aetiology [1] but to date, all attempts to isolate and culture putative pathogens from the skin lesions were unsuccessful, or non-reproducible in later studies [6]. As a consequence, the causal relationship between a specific pathogen and RMS could not be established by traditional methods (i.e., Koch’s postulates). However, culture-independent investigations have been conducted which first suggested an association of RMS with the 16S rRNA gene of a *Rickettsia*-like organism (RLO) [11, 12]. RLO was later identified as a member of the “*Candidatus* Midichloriaceae” family, in the order of *Rickettsiales* [13, 14], and has since been referred to as *Midichloria-*like organism (MLO). Since then, MLO has been consistently reported in correlation with RMS [15–18]. Moreover, contributing to the hypothesis that RMS is caused by a bacterial pathogen, the disease responds to antibiotic treatment [1, 18] and intracytoplasmic, oval Gram-negative-resembling microorganisms have been observed by transmission electron microscopy in tissues of affected rainbow trout from field cases [19, 20].

Even though strong empirical evidence supports the correlation between RMS pathology and the MLO prevalence, all findings remain descriptive. In particular, the possibility that lesions are actually caused by a co-infection or that the MLO is a component cause [11], and not the primary pathogen, cannot yet be ruled out. Both culture-dependent [16] and culture-independent [11, 21] studies of the lesion bacteriome from fish exhibiting clinical signs have been reported before. However, the temporal perspective, which is crucial for the association of a microorganism to distinct phases of a disease by providing observations of its relative abundance oscillations over time, has been absent. A more comprehensive understanding of the bacteria present in and outside lesions during the stages of disease development would help to determine if the MLO is not only present in the skin lesions, but also if it is the only microorganism whose relative abundance correlates with disease status. Moreover, this approach could elucidate possible interplays between different microorganisms in the onset or healing of RMS. Finally, the disease is known to be infectious and horizontally transmissible through cohabitation, and while the MLO was detected in tanks from a field case of RMS [22], longitudinal quantification of the putative pathogen in water and fish during experimental cohabitation has not been comprehensively reported to date.

In this study, we focused on a previously established controlled cohabitation model of RMS [15], and we explored alternative ways of addressing causation [23, 24]. For this purpose, we examined the entire bacterial communities associated with the skin via 16S rRNA gene profiling. In particular, we were interested in: (i) the compositional changes in the skin microbiota over the course of disease progression, (ii) changes in the relative abundance of MLO to further support the hypothesis that it may be the main causative agent of RMS, and (iii) examining the possibility that the disease may be a result of co-infection, through the combined effect of multiple pathogens and/or opportunistic bacteria. Moreover, our approach enabled the characterization of general skin bacterial dynamics during the early RMS pathogenesis and healing stages. To complement the information gathered from the skin, we also described the microbial composition of the water during disease development, to establish a possible effect of RMS on water. Finally, we performed targeted quantification of the MLO in both skin and water along the experiment to corroborate and integrate our findings from microbiome analyses.

## Materials and methods

### RMS cohabitation trial

The study complied with European Regulation [25] on animal research ethics, and was approved by the Animal Experiments Inspectorate, Ministry of Environments and Food under license 2019-15-0201-00159. Rainbow trout eggs (Aquasearch, DK) were disinfected with iodine, then hatched and grown in dedicated Specific Pathogen Free (SPF) animal experimental facilities area in the Section for Fish and Shellfish Diseases (DTU Aqua, Kongens Lyngby, DK). When they reached approximately 50 g of weight, all fish included in the trial were PIT (Passive Integrated Transponder)-tagged in the dorsal muscle anterior to the dorsal fin. For the procedure, fish were anesthetized in benzocaine 100 mg/L (Sigma Aldrich, #E1501) and 2.12 × 12 mm PIT tags (Loligo^®^ Systems, #AB10350) were implanted using dedicated syringes. Once the fish reached an average weight of approximately 140 g, they were transferred to the experimental area in two 180 L cylindrical flow-through tanks. A total of 90 SPF fish were transferred, with 50 placed in the control tank, and 40 in the co-habitation tank. On the following day (0 days post cohabitation), seven additional tagged fish (“seeders”) affected by red mark syndrome were placed in the cohabitation tank.

The seeders were infected by cohabitation 77 days prior the start of the experiment, using an infection model established and propagated as previously described [15, 17, 18, 26–29]. Briefly, as detailed in [15], the model was generated by cohabitation of a batch of rainbow trout presenting RMS lesions, purchased from a Danish recirculating aquaculture system farm declared free of the viral diseases VHS, IHN and ISA, and additionally screened for common fish pathogens (including VHSV, IHNV, IPNV). They were cohabited with naïve rainbow trout, purchased as eyed eggs from a Danish commercial hatchery certified free of the fish-pathogenic viruses IPN, IHN, VHS as well as bacterial kidney disease (BKD), which were iodine-disinfected, hatched and grown in dedicated SPF (specific pathogen free) areas in our experimental tank facilities. RMS-affected rainbow trout infected by cohabitation were subsequently used as “seeders” in the following rounds of cohabitation with new batches of naïve fish in the same manner, to propagate the cohabitation model.

Prior to transfer to the cohabitation tank, RMS infection in seeders was confirmed by macroscopic evaluation and RMS-development scoring (Suppl. Table 1) as described in the following Methods section. The experimental tank conditions were set as follows: water temperature was 12 ± 1 °C, the water exchange rate was fixed to 35 L/hour, and oxygenation was provided via air stones in the tanks for a target saturation above 90%. Both groups were fed 1% body weight daily, with 3 mm EFICO Enviro 920 Advance pellets (BioMar A/S, #590128) administered by clock feeders over the course of 8 h. The fish were fasted for 24 h prior to the end-point sampling. Fish were continuously monitored to ensure a humane end-point, and they were euthanized at the appearance of symptoms such as loss of balance or lesions on the snout (considered caused by mechanical trauma followed by infection). Survival data were collected daily (Suppl. Table 2). As we observed unexpected mortality, routine diagnostic analyses were performed on a subset of mortalities as detailed below, to determine the possible cause of death.

**Fig. 1:**
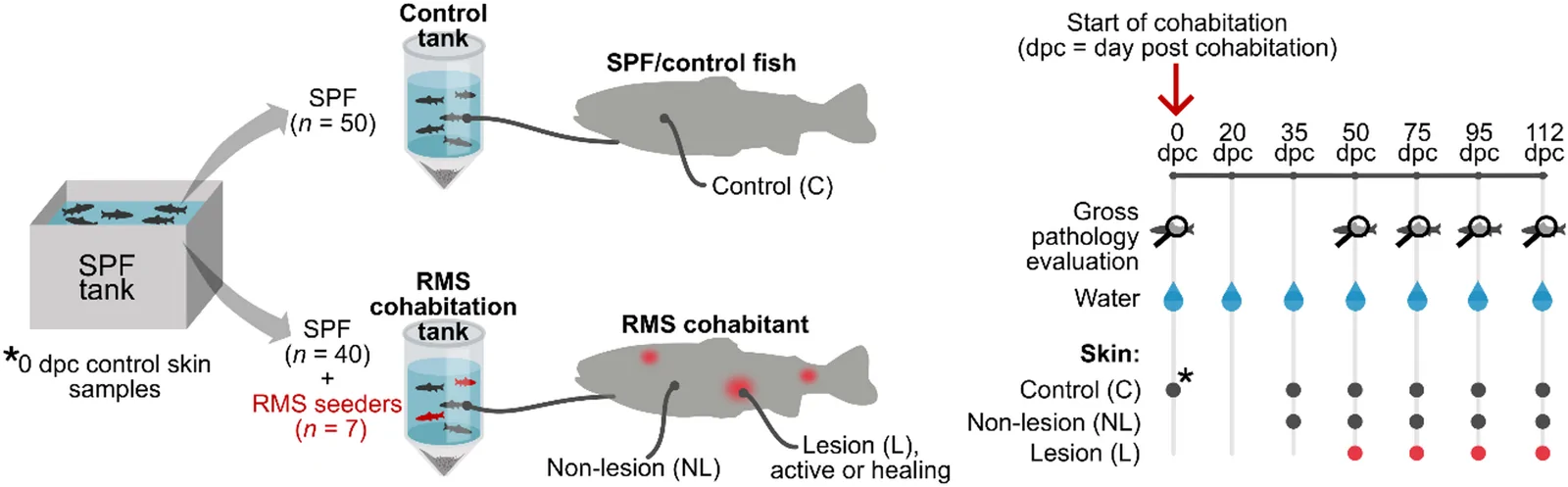
Experimental design: schematic representation of experimental set-up, timeline and sample collection. From the tank holding healthy, specific pathogen free (SPF) rainbow trout, groups of fish were moved in the control tank (*n* = 50) or in the cohabitation tank (*n* = 40). In the cohabitation tank, on 0 days post-cohabitation (dpc), 7 “seeders”, namely individuals affected by red mark syndrome (RMS), were introduced. Control (C) skin samples were collected from either SPF tank (on 0 dpc*) or control tank fish. From RMS-cohabitants, a healthy skin area was collected for the non-lesion (NL) samples; from the same fish, if present (i.e., from 50 dpc) a lesion (L) area, either active or in the healing phase depending on the disease development stage, was collected. Several experimental timepoints were established: 0, 20, 35, 50, 75, 95, 112 dpc. The experimental timeline on the right depicts when gross pathology evaluation, water sampling, or skin sampling were performed. The illustration was generated using Inkscape v1.2

In Fig. 1, we provide a schematic representation of the experimental design and sampling strategy.

### Visual inspection and scoring of macroscopic changes

On days post-cohabitation (dpc) 0, 50, 75, 95, 112, all individuals from the control and RMS tank were anesthetized in benzocaine 100 mg/L (Sigma Aldrich, #E1501), measured, weighted, and pictures were taken for visual inspection and macroscopical RMS pathology evaluation (Suppl. Table 3) through the scoring system previously defined in [18] and reported in Table 1.

**Table 1:** RMS development gross pathology scoring system used to evaluate fish during the experimental cohabitation trial

RMS score	Description	Skin sample type group
No lesion	Complete absence of lesions, normal colour of skin, scales are present.	Non-lesion
Mild lesion	Mild oedema, opaque to light pink colour, scales are present.	Active lesion
Moderate lesion	Moderate oedema, pink to red colour, scales are present or mild resorption of scales.	
Severe lesion	Severe oedema, bright red colour, mild to severe scale resorption.	
Early healing stage	Mild to moderate oedema, dull red to brown colour, variable state of scales.	Healing or healed lesion
Late healing stage	No oedema, brown to normal colour, variable state of scales.	
Healed	No oedema, normal colour of the skin, scales present. Similar to the ‘No lesion’ category, with the difference that lesions were previously observed.	

### Histological examination of skin samples

On 75 dpc, additional skin samples were aseptically excised and collected from cohabitants (active lesion samples) and control fish (control skin samples) for the purpose of histological examination and RMS pathology confirmation (*n* = 5 per group). The excised skin layer was 0.5 cm to include epidermis, dermis, hypodermis and muscle. Samples were stored in 10% neutral buffered formalin (4% formaldehyde). Subsequently, samples underwent standard steps of fixation, dehydration, embedding in paraffin, cutting, mounting and staining with H&E. Images were acquired using a Leica DM4000B microscope and the software Application Suite v4 (Leica Microsystems, DK). Histological images were stitched together in composite images, using consistent scaling and alignment, using Inkscape v1.2.

### Diagnostic examinations

As RMS does not usually cause mortality in fish, routine diagnostic tests were performed to assess the possible cause of death for a subset of moribund (euthanized) fish. Head kidney and gills from a subset of mortalities (cohabitants *n* = 7, controls *n* = 1) were tested for the presence of the following bacterial species: MLO, as the primary candidate pathogen in our model; “*Candidatus* Branchiomonas cysticola” [30], selected because of its high relative abundance found in the skin in the present study and in internal organs in a previous 16S rRNA gene sequencing study using moribund fish from the RMS infection model (unpublished), and due to its detection in internal organs of Atlantic salmon affected by ulcerative skin lesions [31]; *Renibacterium salmoninarum* [32], selected due to a previous suspicion of contamination in our experimental tank facilities; and “*Candidatus* Piscichlamydia salmonis” [33], as part of our efforts to increase our understanding the presence of this bacterium in the common microbiome of rainbow trout and its role as a potential pathogen (Suppl. Table 4). Not only gills and kidney of dead individuals, but also those of randomly sampled rainbow trout on 50 dpc (controls *n* = 5, RMS cohabitants *n* = 5) were included in the diagnostic examinations (Suppl. Table 5). Tissue samples were homogenized in 220 µL of sterile PBS on a Tissuelyser II (Qiagen) at 25 Hz for 3 min and subsequently extracted using an IndiMag extraction system (Indical Bioscience) with the IndiMag Pathogen Kit (#SP947457, Indical Bioscience). Samples were then tested by qPCR using 5 µL of template DNA, 7.5 µL of 2X Luna^®^ Universal Probe qPCR Master Mix, forward and reverse primers 0.4 µM (0.8 µL) with probe 0.2 µM (0.4 µL), and RNAse-free water to a reaction volume of 20 µL. qPCR reactions were carried out using a CFX Opus 96 thermocycler (Bio-Rad), and raw data were retrieved and analysed using the CFX Maestro software (Bio-Rad). The samples were also subjected to MLO detection using the qPCR assay described below. In addition, a random subsample of dead fish from the RMS cohabitation tank (*n* = 3) was tested by plating of internal organs (brain, kidney) on tryptone yeast extract salts (TYES) agar, to culture for other bacterial pathogens such as *Flavobacterium psychrophilum*, which had previously been suggested as a potential contributor to RMS in an early study [34].

### Skin and water samples for the study of bacterial community composition and MLO quantification

At the start of cohabitation, six SPF rainbow trout were collected from the original tank where all SPF fish for the experimental trial were grown. This was considered as the “baseline status” for SPF fish not subjected to cohabitation (0 dpc). On 35, 50, 75, 95 and 112, five randomly selected fish from each experimental group (control or cohabitation condition) were euthanized using a lethal dose of benzocaine (Sigma-Aldrich). Skin tissue samples were collected immediately after euthanasia as detailed previously [35]. Briefly, skin samples were collected from the euthanized fish as follows: with a sterile scalpel an approximately square incision of 15 × 15 mm was made through the skin on the flank. With toothed sterile tweezers, the skin was lifted and detached from the muscle layer beneath. The skin sample was placed in a sterile 2 mL Eppendorf tube, and immediately snap-frozen on dry ice. For cohabitants, when lesions were present, both lesion and non-lesion skin areas were sampled from different regions of the same flank to be analysed separately, as shown in Fig. 1.

On days 0, 20, 35, 50, 75, 95, 112, water samples were collected in four replicates from the control and cohabitation tanks using autoclaved glass bottles and stored at 4 °C until filtration (maximum 24 h), then processed as detailed in [35]. Briefly, a vacuum-filtration system consisting of a vacuum pump connected to bottle top filtration units (Nalgene) on autoclaved glass bottles was set up in a LAF-bench to avoid contamination. Up to 500 mL of water (or until membrane clogging) was filtered through hydrophilic PVDF filter membranes of 47 mm diameter and a pore size of 0.22 μm (#GVWP04700, EMD Millipore Durapore). Each filter was sectioned and placed in a sterile 2 mL Eppendorf tube, and immediately snap-frozen on dry ice.

### Total nucleic acid extraction

For nucleic acid extraction, MagMAX Microbiome Ultra Nucleic Acid Isolation Kit (Applied Biosystems) was used in conjunction with the automated extraction system KingFisher Flex (Thermo Fisher Scientific) as previously described [35]. The same kit was used for water and skin samples, with minor modifications in the pre-processing of the different sample types.

For water, the entire filter was added zirconia beads and 800 µL of lysis buffer, then homogenized on a Tissuelyser II (Qiagen) at 25 Hz for 2 min 30 s.

Skin tissue samples were similarly homogenized with zirconia beads, with the addition of a 0.5 cm sterile stainless-steel bead (Qiagen) and 1 mL of lysis buffer; subsequently, they were diluted 1:1 by transferring 400 µL of homogenate to a new tube containing 400 µL of lysis buffer.

All samples were spun briefly in the centrifuge, and 9 µL of lysozyme solution (80 mg/mL) was added to each sample. Samples were incubated at 37 °C for 1 h. Subsequently, 500 µL of homogenized samples were carried on to extraction as per manufacturer’s instructions. Negative controls of extraction for both skin (reagents only, and reagents with stainless steel bead) and water (reagents only, and reagents with unused PVDF filter membrane) were included, as well as an aliquot of ZymoBIOMICS™ Microbial Community Standard (#D6300, Zymo Research). Nucleic acids were eluted in 100 µL of elution buffer and immediately aliquoted and stored at -80 °C until further processing.

### Quantification of MLO in skin and water samples using qPCR

Previously developed qPCR primers, designed to generate a 127 bp long amplicon, were selected for MLO detection [13]. Primer sequences are reported in Table 2.

**Table 2:** Primer sequences used in the present study

Oligonucleotide name	Gene	Sequence (5’-3’)	Reference	Use
*MLO-fw*	16S rRNA gene of MLO (GenBank: EU555284.1)	GCGGTTATCTGGGCAGTC	Cafiso et al., 2016	MLO molecular quantification by qPCR
*MLO-rv*		TGCGACA**C**CGAAACCTAAG		
515 F	16S rRNA gene hypervariable region V4	GTGCCAGCMGCCGCGGTAA	Caporaso et al., 2011	Microbiome profiling by amplicon sequencing
806R		GGACTACHVGGGTWTCTAAT		

In the oligonucleotide sequences reported here, a one-nucleotide (bold) typo in the reverse primer from Cafiso et al., 2016 was corrected (incorrect version with typo: TGCGACACGAAACCTAAG)

Next, 2x Brilliant II SYBR Green qPCR Master Mix (#600828, Agilent Technologies) was used following the manufacturer’s instructions. Briefly, 12.5 µL of SYBR Master Mix was mixed with 1 µL of each primer oligonucleotide (400 nM). Nuclease-free water and DNA template were added for a final reaction volume of 25 µL. Specifically, for skin DNA samples, 4.5 µL of DNA template (previously diluted 1:10 in nuclease-free water) was used for each reaction; for water DNA samples, 3 µL of undiluted DNA template was used. Negative controls for each experimental stage (including extraction reagents and no template control) were also analysed in each qPCR run. An artificial DNA control (g-Block, IDT) for MLO 16S rRNA gene was used to create a standard curve. The artificial DNA control dilutions were also used as inter-plate calibrators, to account for inter-run variability by aligning threshold settings and thus allowing comparison between qPCR runs. All qPCR reactions were carried out using a CFX Opus 96 thermocycler (Bio-Rad). The program was set to an initial denaturation of 10 min at 95 °C followed by 40 amplification cycles of 30 s at 95 °C, 60 s at 60 °C, followed by melting curve analysis: 60 s at 95 °C, cooling to 55 °C, then gradual heating to 95 °C with fluorescent acquisition at incremental temperature steps (0.5 °C/step). Raw data were retrieved and analysed using the CFX Maestro software (Bio-Rad). Samples with a quantification cycle (Cq) under the limit of detection and a multi-peak or incoherent melting curve (suggesting primer dimerization or unspecific signal) were marked as negative. For graphical representation, MLO copies were normalized based on the total DNA ng that were used as a template for each qPCR reaction (Suppl. Table 6) and log transformed (log_10_, adding a pseudo-count of 1). In the case of water, the number of copies was normalized based on the volume (mL) of analysed water instead (Suppl. Table 7).

### Library preparation and 16S rRNA gene amplicon sequencing

DNA extracted from skin and water samples was sequenced by Novogene Co., Ltd. through 16S rRNA gene metabarcoding. In addition to negative and positive controls of extraction, an aliquot of ZymoBIOMICS™ Microbial Community DNA Standard (#D6305, Zymo Research) was included in the sample set to sequence. The V4 universal primers 515 F-806R [36], reported in Table 2, were used for PCR amplification, with the addition of barcodes for library multiplexing.

Library preparation was performed using the NEBNext^®^ Ultra™ II DNA Library Prep Kit for Illumina (New England Biolabs, Cat. No. E7645) as follows: PCR amplification was performed using 15 µL of Phusion^®^ High - Fidelity PCR Master Mix (New England Biolabs), 0.2 µM of primers (forward and reverse), and approximately 10 ng template DNA, and thermal cycling conditions were set to 98℃ for 1 min for initial denaturation, followed by 30 cycles at 98 °C for 10 s, 50 °C for 30 s, 72 °C for 30 s, and final elongation at 72 °C for 5 min. The quality control (QC) of the PCR products was conducted with a capillary electrophoresis system (Agilent 5400). Samples lacking the expected ~ 300 bp amplicon products were designated as QC fails. Amplicons were purified using magnetic beads. Equimolar amounts of PCR products from each sample were pooled, size-selected, end-repaired and A-tailed, and further ligated with Illumina adapters. Libraries were sequenced on the Illumina NovaSeq 6000 platform to generate 250 bp paired-end raw reads, with a minimum of 30 K reads/sample.

### 16S rRNA gene amplicon data analysis

The bioinformatic analysis was performed starting from demultiplexed, raw paired-end (PE) reads containing adapters (Illumina Universal Adapter—AGATCGGAAGAG), barcodes and primers. CutAdapt v4.8 [37] was used to trim the primers, setting un-anchored primer sequences and a minimal length of 20 nt (-m 20). Using DADA2 v1.30.0 [38], truncation was performed based on quality profiles (truncLen = c(160,160)), and reads were further truncated and filtered according to their quality scores (maxEE = c(2,2), truncQ = 2). Forward and reverse reads were dereplicated and sample inference was performed based on their error profiles. Forward and reverse reads were then merged and a sequence table was constructed. Chimeric amplicon sequence variants (ASVs) were removed *de novo* using default parameters. All samples retained > 75% of their reads after pre-processing (Suppl. Table 8).

Taxonomic assignment of ASVs was performed using the *assignTaxonomy* function of the DADA2 package against the SILVA database, release 138.2 [39]. The output of DADA2 was manipulated for further analysis using phyloseq v1.42.0 [40] and SQMtools v1.7.0 [41]. Only bacterial sequences were retained by filtering out Mitochondria and Chloroplast sequences. The most abundant ASV (ASV_0001, Suppl. Table 9) was only classified at the Superkingdom rank (Unclassified Bacteria). Basic local alignment (BLASTN) [42] against the NCBI nucleotide collection (nt) database revealed 100% identity with an *Oncorhynchus mykiss* mitochondrial sequence (GenBank: MT667254.1). The ASV was therefore excluded, resulting in most skin samples’ read counts dramatically decreasing while the water samples still retained > 90% of the reads (Suppl. Table 10). One sample was discarded because of insufficient read count after elimination of the *O. mykiss* mitochondrial sequence (2278 reads), based on the rarefaction curve. Five outlier skin samples were filtered out based on the aberrant number of observed features and composition profiles (Suppl. Figure 1). The skin dataset was rarefied to an even sample size of 9756 reads. Water samples were instead rarefied to the lowest read/depth in the water dataset (35658 reads). Rarefaction curves were plotted for both datasets before and after rarefaction (Suppl. Figure 2), according to observed ASV counts or Shannon diversities calculated using vegan v2.6.8 [43].

Two ASVs (ASVs 14 and 32, Suppl. Table 9), considered of interest because of their high relative abundance in the skin at peak RMS pathology, were subjected to phylogenetic analysis including selected sequences from [44]. Sequences were aligned using Muscle v5.1 [45], and a maximum likelihood tree was constructed with PhyML v3.3 [46] using the MKY85 + G model with 100 bootstrap replicates.

Ordination was performed on rarefied ASV counts using PCA based on Robust Aitchison distances with gemelli v0.011 [47]. ASVs reported on the ordination plots were selected based on their distance from the centre (ASV coordinates > 0.05 or < -0.05 on PC1 and PC2) and an overall mean (across all samples) relative abundance threshold of 0.5%. ALDEx2 v1.36.0 [48] was used to perform differential abundance analysis, using the non-rarefied read counts – as ALDEx2 was shown not to be impacted by rarefaction or prevalence threshold filtering [49] – as input data. The *aldex.clr* centred log ratio transformation function was used with 170 Dirichlet Monte-Carlo Instances. The *aldex.glm* function was used with a model matrix built on the skin sample category variable and the cohabitation day covariate.

### Statistical analysis

R Statistical software v4.4.1 [50] was used for all statistical analyses and plots. Kaplan-Meier survival probability estimation analysis was performed using the package ‘survival’ v3.7.0 [51], considering only cohabitants (not RMS seeders). All graphical representations were performed using ggplot2 v3.5.1 [52]. All scripts used to obtain the presented results are available on GitHub (https://github.com/zrngiulia/Red-mark-syndrome-16S).

## Results

### Naive individuals successfully developed RMS pathology during cohabitation

**Fig. 2:**
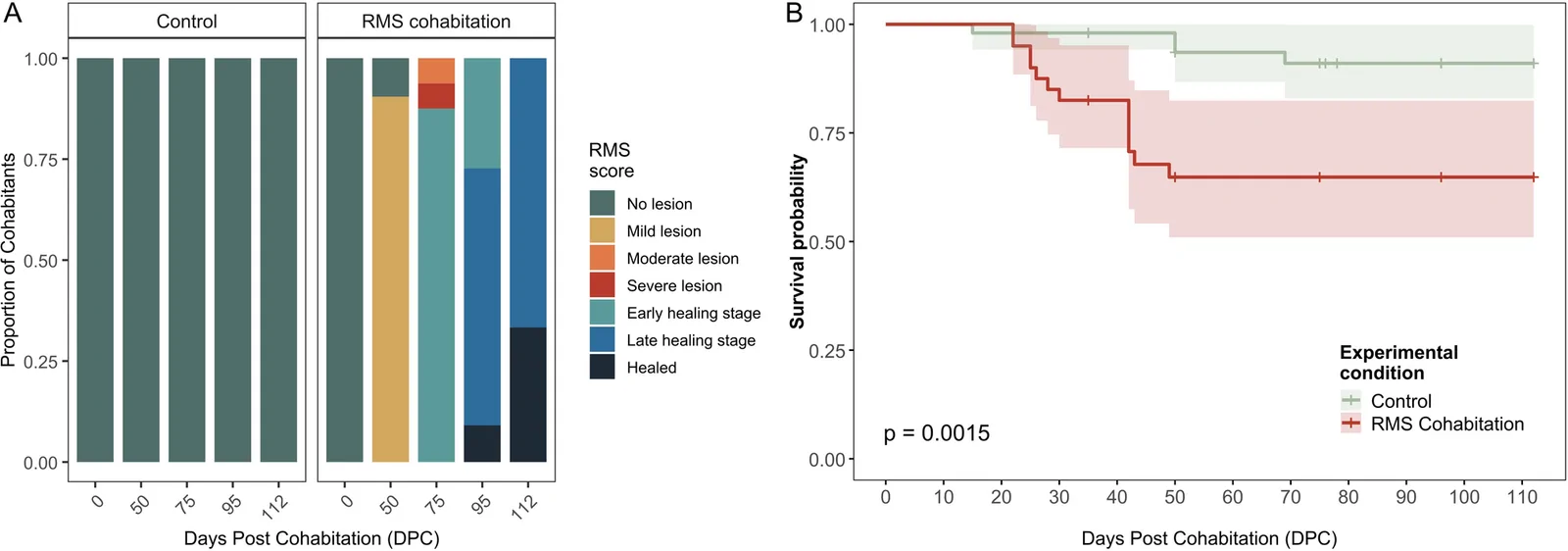
Cohabitation with seeders affected by red mark syndrome (RMS) resulted in the development of RMS in cohabitants, and in increased mortality. **(A)** Proportion of rainbow trout assigned with RMS development scores over time. The stacked barplots represent the proportions of individuals which were assigned each score, in the control or in the cohabitation tank, upon evaluation for RMS development from ‘No lesion’ to ‘Healed’, over the time of cohabitation trial. Data are represented as proportions over the total for ease of representation and group comparison. RMS seeders are not included in the plot. **(B)** Survival curves for infected cohabitants and non-infected controls. Kaplan-Meier survival analysis shows a significant increase in the survival probability of the control group (green) with respect to the cohabitants of RMS-affected fish (red) (*p* = 0.0015). Data are shown as survivors over time. Ticks in the curves represent censored individuals. RMS seeders are not included in the calculation of survival probability and curves

During the course of our experimental trial, naive rainbow trout developed clinical RMS following cohabitation with RMS-affected individuals termed “seeders”. Seven seeders were introduced in the tank on 0 dpc, presenting either mild (*n* = 4), moderate (*n* = 1), severe (*n* = 1) or early healing lesions (*n* = 1). Except for one seeder which reached a humane endpoint on 16 dpc, from 50 dpc all seeders were in the healing stages of RMS. Mild lesions, typically on the flanks but also on more ventral and dorsal skin areas, were visible on most cohabitants by 50 dpc. At 75 dpc, a minor proportion of cohabitant fish still had active lesions, but most individuals appeared to be recovering, indicating that the peak of disease had occurred between 50 and 75 dpc. At later timepoints (95 and 112 dpc), all cohabitant fish presented lesions in early or advanced healing stages (Fig. 2A).

RMS is not known to be associated with an increase in mortality rates in rainbow trout. However, over the course of the experimental trial, a proportion of rainbow trout in both control and cohabitation tanks reached a humane end-point and were euthanized before experiment termination. A total of thirteen RMS cohabitants (32.5%) and four fish in the control tank (8.0%) reached a humane end-point. As such, the survival probability (Fig. 2B) was significantly higher in the control condition compared to the RMS cohabitation condition (*p* = 0.0015, Kaplan-Meier survival probability estimation). All mortality in cohabitants occurred between 22 and 49 dpc, with a mortality peak at 34 dpc. Diagnostic examinations via qPCR confirmed the presence of “*Ca*. Branchiomonas cysticola” in all gills (Cq = 20.48 ± 1.54) and in 6 out of 7 kidneys (Cq = 33.52 ± 2.51) of examined dead individuals. Interestingly, the kidneys of a subset of examined dead cohabitants (*n* = 2) were also positive for MLO (Cq = 33.33 ± 0.16) (Suppl. Table 4). Diagnostic analyses were negative for the presence of other bacterial pathogens tested for using qPCR, and no colonies were isolated from culture media.

Diagnostic examinations via qPCR were also carried out in randomly sampled rainbow trout (controls *n* = 5, RMS cohabitants *n* = 5) on 50 dpc. “*Ca*. Branchiomonas cysticola” was detected in all gill samples, however with higher loads in the gills of RMS cohabitants (Cq = 25.70 ± 2.17) than in those of controls (Cq = 35.42 ± 1.78). The MLO was not detected in any of the control rainbow trout tissues. It was however detected in the gills of two RMS cohabitants, and in all kidneys in this group (Cq = 33.75 ± 1.57). Notably, a concomitant low-level detection of “*Ca*. Branchiomonas cysticola” (Cq = 39.23 ± 0.87) was observed in the same kidney samples where the MLO was also detected (Suppl. Table 5).

**Fig. 3:**
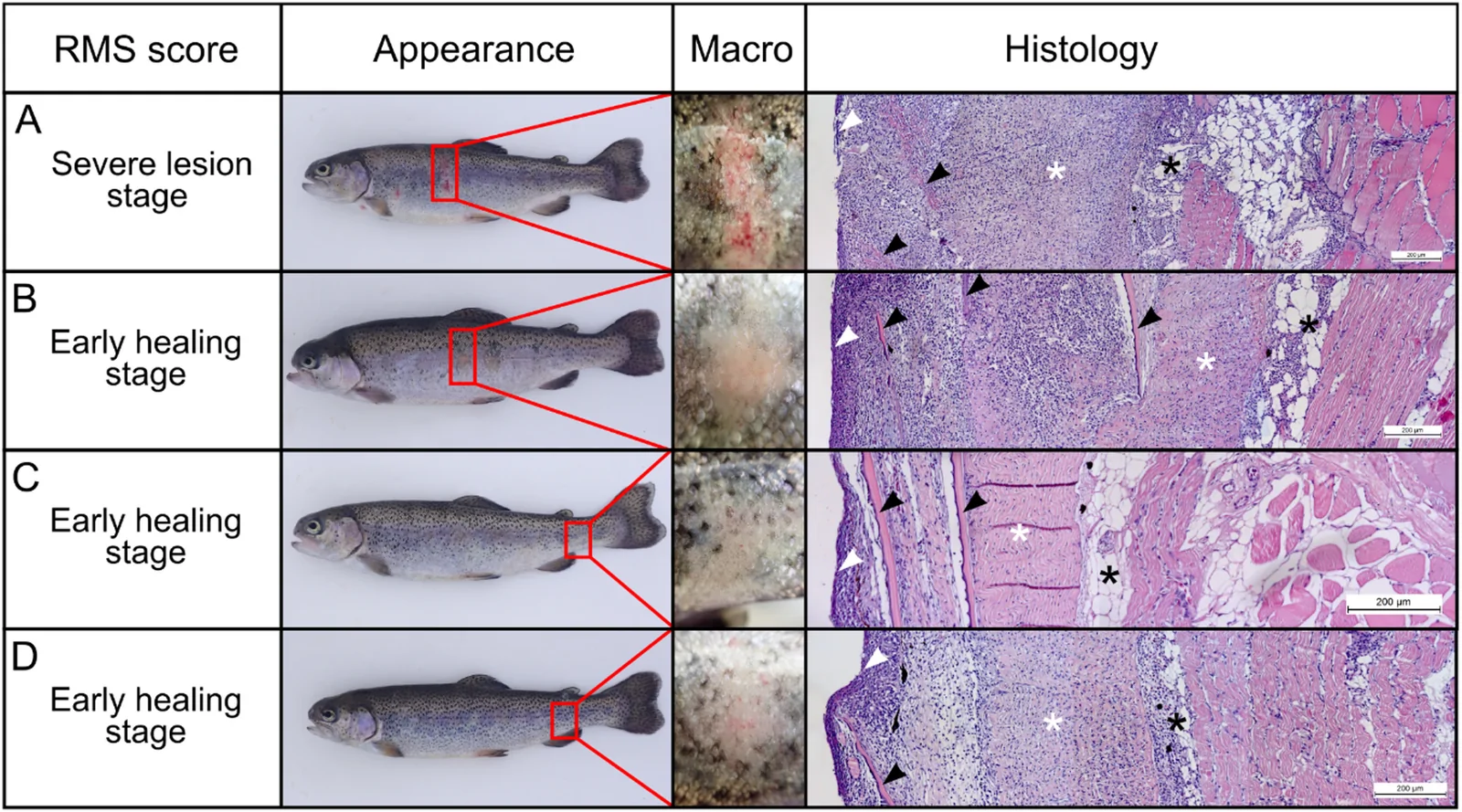
Macroscopical appearance of fish, lesions, and histological features of skin lesions at 75 dpc. Histologically, inflammation and cellular infiltration involve skin layers from epidermis (left) to the hypodermis and muscle layers (right), to varying degrees and according to RMS development score. Composite histological images were manually stitched together using Inkscape v1.2. **(A)** Cohabitant with active lesions, with an assigned RMS development score “Severe lesions”. The epidermis is partially missing. Scale pockets are visible in the stratum spongiosum, but scales are absent. Extensive and severe cellular infiltration is present in all skin layers, including stratum spongiosum, compactum, hypodermis and down to the muscle. **(B)** Cohabitant in the early healing stage. Partially resorbed scales are visible in the scale pockets. Cellular infiltration is present in all skin layers, to a lesser degree than in the active lesion. **(C)** and **(D)** show cohabitants in the early healing stage, which had never progressed through a “severe” stage but passed directly from mild lesions to the healing stage. Macro lesion pictures show a milder pathology, and also histologically, skin layers are less affected; Scales are present (black arrowheads), and inflammatory infiltration, despite visibly involving all skin layers, is also milder, however with congested blood vessels **(D)** remaining evident near the epidermis. White arrow heads: epidermis. Black arrow heads: scales and scale pockets. White asterisks: stratum compactum. Black asterisks: hypodermis. The scale bar corresponds to 200 μm

At 75 dpc, lesion skin areas sampled for histopathological analysis displayed the typical signs of RMS, confirming the successful infection of the individuals. In particular, the severe lesion showed complete absence of scales, and extensive cellular infiltration in all skin layers from epidermis down to the muscle (Fig. 3A). Lesions from cohabitants in the early healing stage displayed milder cellular infiltration, and scales were present (Fig. 3B, C, D). As expected, the skin of control individuals did not show any signs of RMS or skin disease.

### MLO 16S rDNA copies are most abundant at peak of RMS pathology

**Fig. 4:**
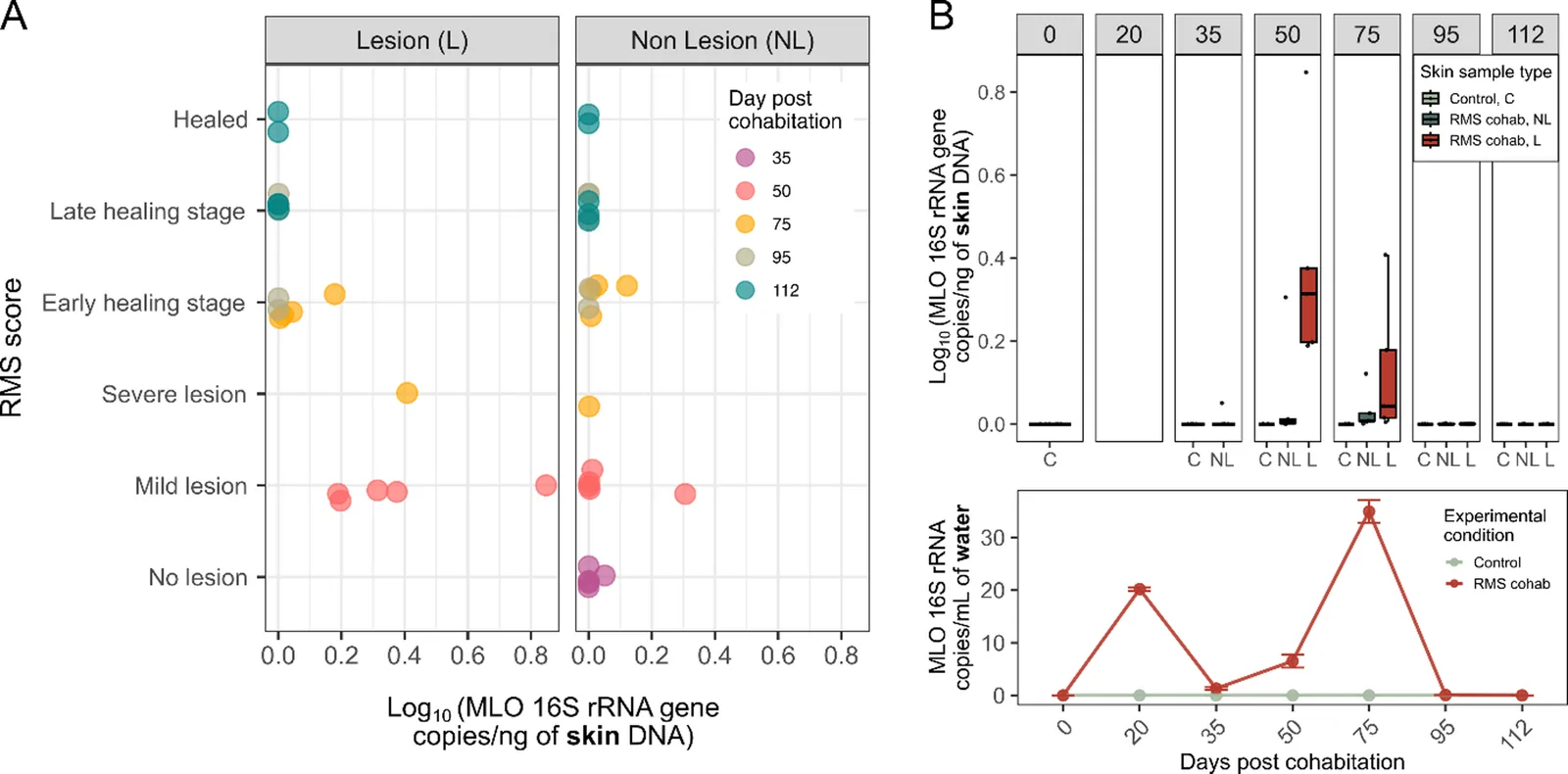
*Midichloria*-like organism (MLO) quantification in the fish skin and rearing water, stratified by skin sampling site, red mark syndrome (RMS) score of the fish, or timepoint. **(A)** Lesion or non-lesion skin samples of RMS cohabitants are shown, with MLO quantification results expressed as 16S rRNA gene copies/ng of DNA. On 35 dpc, all fish were scored as “no lesion”, and therefore lesion samples were not present. Subsequently, RMS cohabitants underwent the progressive stages of disease, from mild to severe lesion, and the different healing stages. MLO quantification reflects the disease development status of the fish, and the quantity of MLO was more generally higher in lesions compared to non-lesion skin areas. Control skin samples from the control tank are not shown. **(B)** MLO quantification in skin and water samples over time. The boxplot **(top)** represents MLO 16S rRNA gene quantification in skin samples (copies/ng of DNA, logarithmic (log_10_) scale) over time, with a peak at 50 dpc in lesion (L) skin areas. Data are grouped in control (C), lesion (L) and non-lesion (NL) skin samples (*n* = 5). The control samples (*n* = 6) at 0 dpc were collected from the original SPF tank. Results are plotted as median with interquartile range, and data are shown after logarithmic transformation (log_10_) with the addition of a pseudocount of 1. The lineplot **(bottom)** represents the MLO load in the water of control or cohabitation tank (16S rRNA gene copies/mL). MLO quantity is shown over time (dpc), with 2 separate peaks for the RMS cohabitation condition. In the control condition, MLO was not detected. Each dot represents the mean (*n* = 4 replicates of filtration) ± standard error (SE)

Whereas no MLO was detected in the skin of negative control fish, the bacterium was detected by qPCR in skin samples from RMS cohabitants from 35 up to 112 days post-cohabitation. MLO was found to be generally more abundant, according to qPCR-based quantification, in RMS cohabitant fish with active lesions compared to those in the preclinical stage (no lesion) or healing stages, and more abundant in lesions compared to non-lesion areas (Fig. 4A). From a temporal perspective, the highest quantity of MLO was detected at 50 dpc, when all sampled fish presented with mild active lesions. MLO was also present but as lower read counts relative to earlier time points in lesions at 75 dpc, when only one sampled fish showed severe lesions and the rest were already in the early healing phase. The quantity of MLO then further declined during the healing stages and when fish were healed (from 95 to 112 dpc), as shown in Fig. 4B.

In addition to skin samples, we detected and quantified the bacterium in water by analysing the environmental DNA (eDNA) using the same qPCR assay. The MLO was detected in water from the very early stages of the cohabitation trial (20 dpc) up to the healing phase of RMS (95 dpc). We observed two distinct peaks of MLO levels in water, a first at 20 dpc, and a second at 75 dpc (Fig. 4B). This is consistent with an initial shedding from seeder fish (*n* = 6), which at this stage presented lesions at different healing stages. The second, more pronounced peak at 75 dpc is consistent with shedding from the cohabitant fish (*n* = 16), which at this stage were almost all in the early healing stage. At the end of the experiment, at 112 dpc, MLO was completely undetectable by qPCR in water. Finally, and importantly, MLO could not be detected in the control tank at any time point.

### Metabarcoding sequence analysis

PCR targeting the 16S rRNA gene did not yield an amplification product in any of the negative controls (Suppl. Figure 3). Positive controls of extraction and PCR amplification successfully captured the theoretical 16S rRNA gene microbial composition of the mock community (Suppl. Figure 4). Concerning skin and water samples, 16S rRNA gene sequencing yielded a minimum of 34,267 and a maximum of 96,334 raw reads/sample. After filtration, reads merging and chimeric sequences removal, the final number of reads per sample ranged from 32,882 to 89,222. After filtering non-bacterial sequences and rarefaction, a total of 1,165 ASVs were obtained from skin samples and 2,144 from water samples.

### Bacterial composition in water samples

**Fig. 5:**
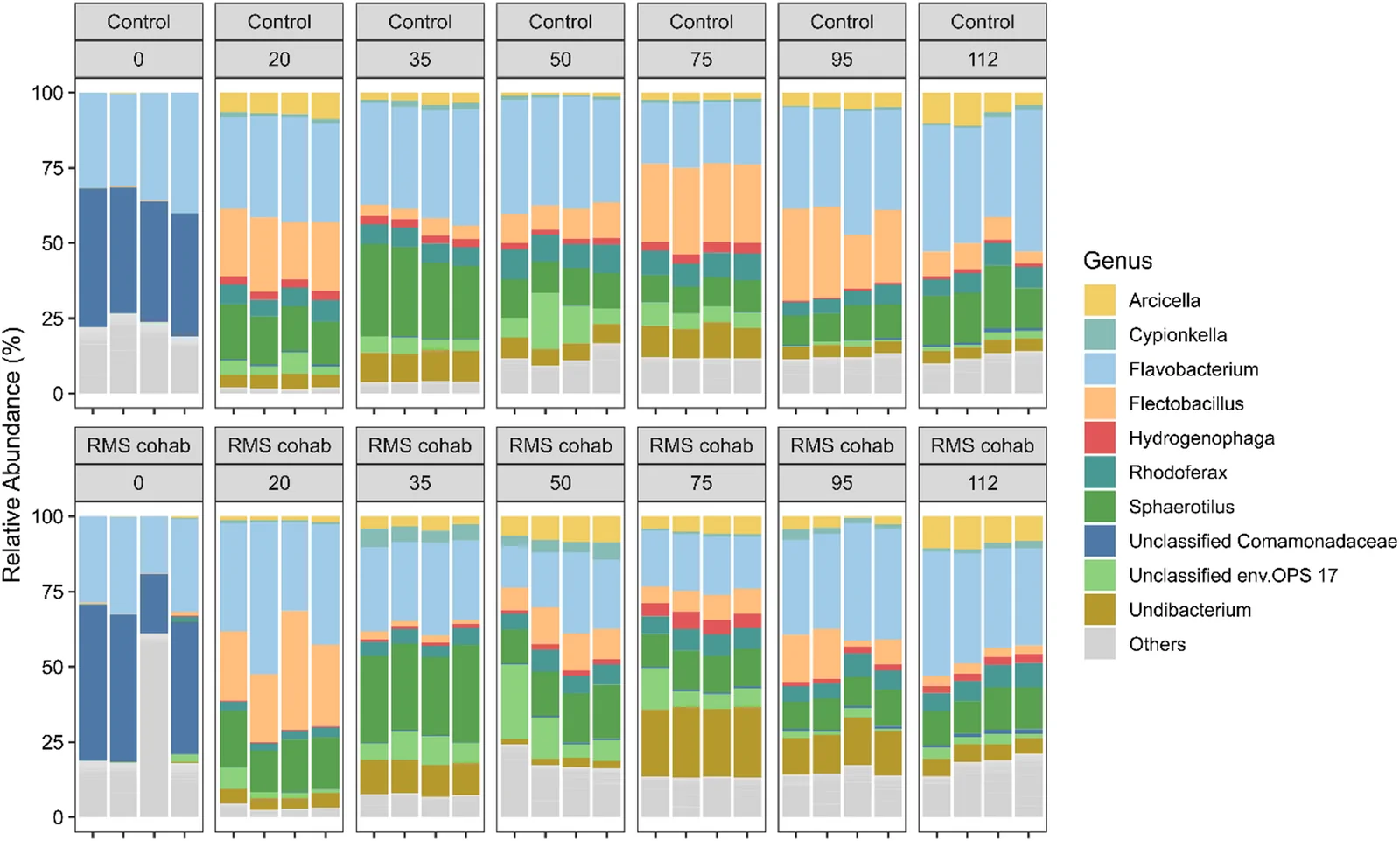
Microbial composition of water samples over time. The stacked barplots represent the relative abundance (%) of the top 10 most prevalent genera. The remaining taxa are grouped and shown as “Others”. Control tank water samples are on top, while RMS cohabitation water samples are on the bottom. 4 technical replicates of water filtration are shown stratified by day post cohabitation (0 to 112 dpc)

Microbiome analysis of water samples revealed a similar bacterial community composition in the control and cohabitation tanks at the start of cohabitation (0 dpc), with the most prevalent taxa being *Flavobacterium* and a group of unclassified *Comamonadaceae* (Fig. 5). At subsequent sampling points, cohabitation and control tanks showed a switch towards a more diverse composition with an increase in the relative abundance of *Flectobacillus*, *Sphaerotilus*, *Arcicella*, *Rhodoferax*, *Undibacterium* and a group of unclassified *env. OPS 17* genera.

**Fig. 6:**
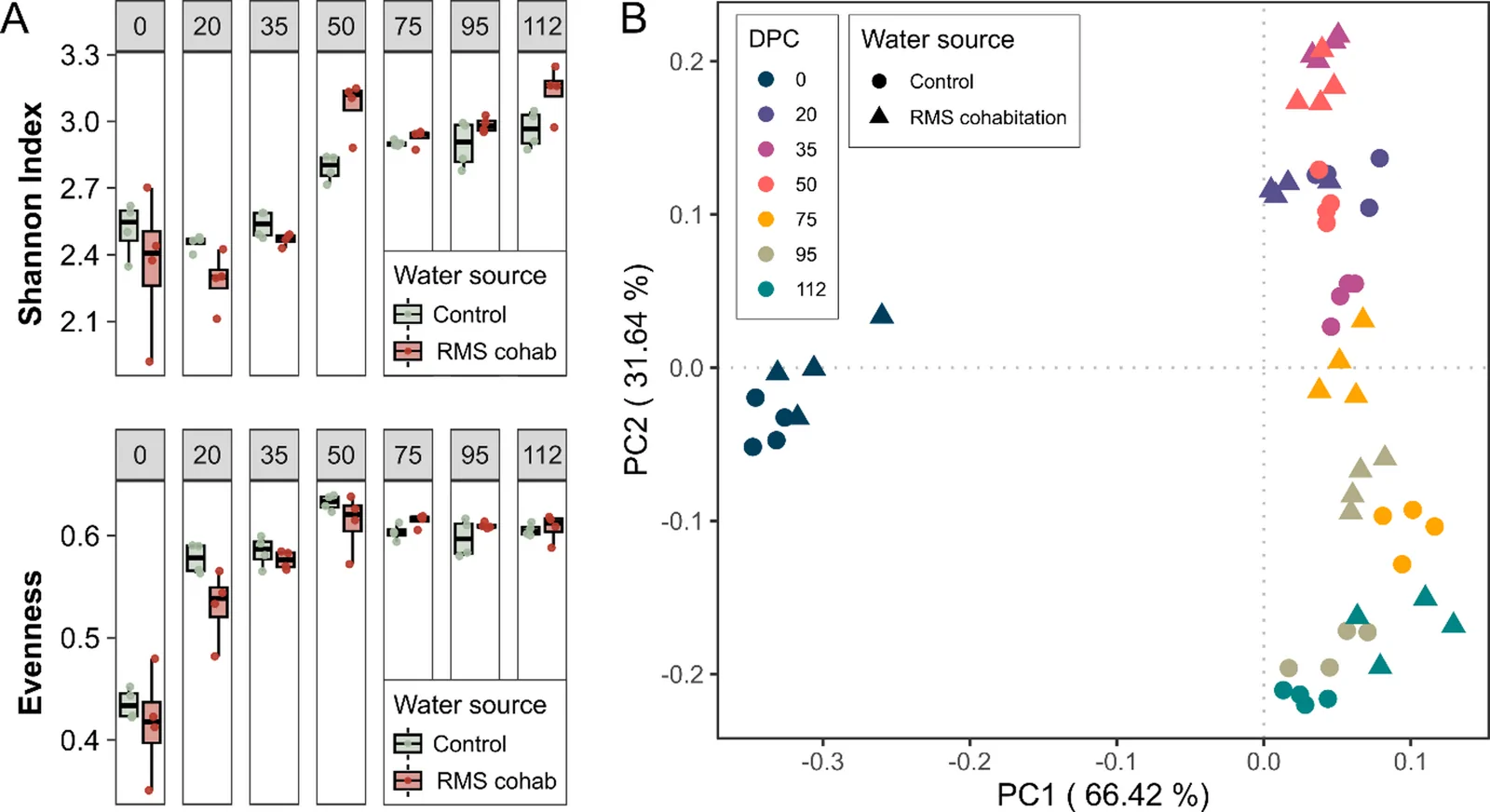
Alpha and beta diversity metrics of water samples.** (A)** Boxplots represent the alpha diversity indexes, Shannon’s or Pielou’s, of each water sample (*n* = 4 technical replicates of filtration) divided by experimental condition and sampling time point. Data are plotted as median with interquartile range. **(B)** Beta diversity metrics of water samples. Robust Aitchison’s distance-based Principal Component Analysis (RPCA) ordination plot, ASV-level analysis. Technical replicates of water filtration (*n* = 4) are plotted as separate data points. Samples are coloured by sampling time point, and the condition (control or RMS cohabitation) is differentiated by shape

This increase in complexity over time was also observed as an increase in alpha diversity (Shannon, Pielou) from dpc 0 to dpc 112 (Fig. 6A). Beta diversity was visualized using a Robust Aitchison distance-based PCA plot, with principal components 1 and 2 accounting for 66.4% and 31.6% of variation in water microbial communities, respectively (Fig. 6B). Water samples first clustered by groups of technical replicates (*n* = 4), indicating a low variation among these. Although water samples from the two tanks overlapped on 0 and 20 dpc, a tank effect was also visible from 35 dpc, with the water from the cohabitation and control tanks diverging from each other at each subsequent sampling point. However, the major driver of variation was time. Indeed, samples grouped primarily according to sampling point, with a marked change from 0 dpc to all later sampling days, and the microbial composition of control and RMS-cohabitation tank changing in the same direction along the trial (Suppl. Figure 5). Interestingly, the “*Ca.* Midichloria” genus was not detected by 16S rRNA gene amplicon sequencing in any of the analysed water samples.

### Skin bacterial community complexity increases over time, with the exception of skin from fish with active lesions

**Fig. 7:**
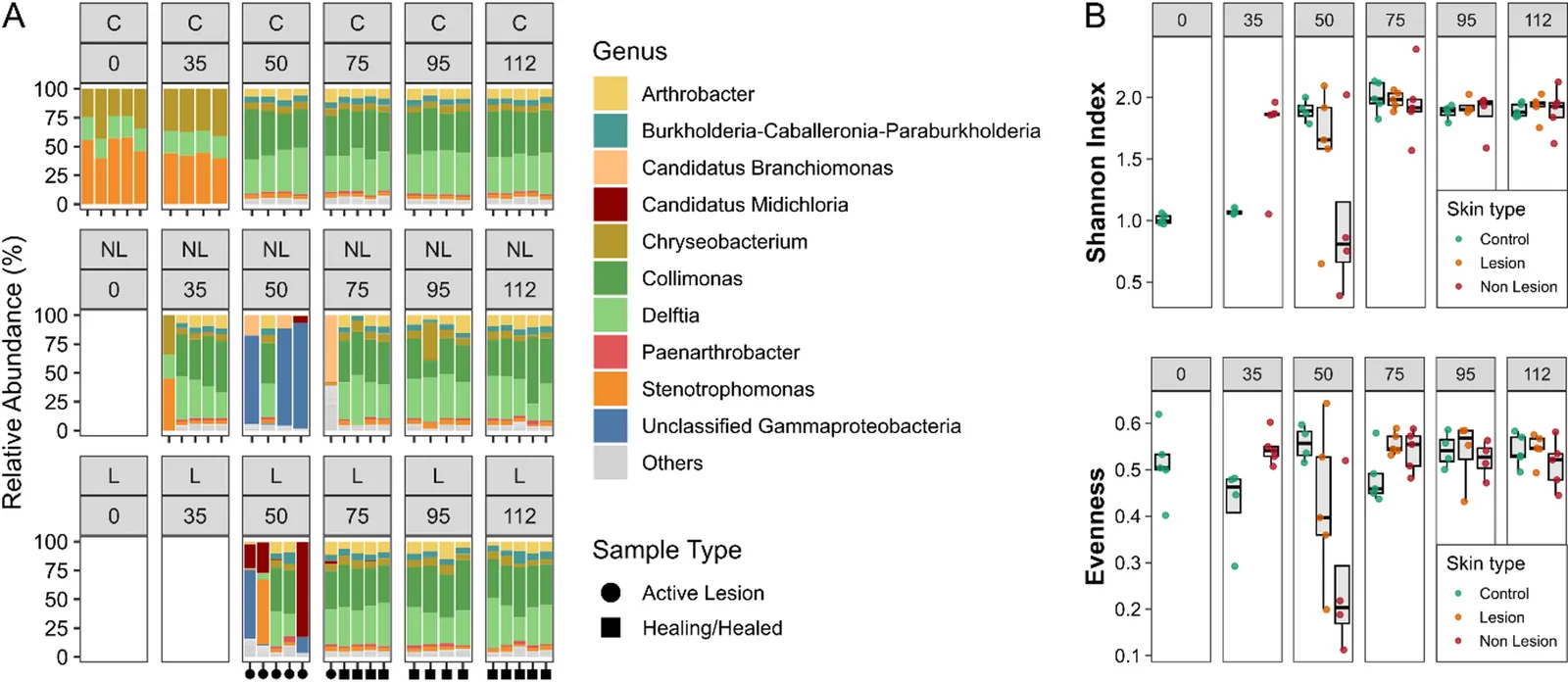
Microbial composition and alpha diversity of skin samples over time. **(A)** The stacked barplots represent the relative abundance (%) of the top 10 most prevalent genera. The remaining taxa are grouped as “Others”. Control samples (C) are on top, while RMS cohabitation samples are on the bottom, divided into non-lesion (NL) and lesion (L) skin areas. The type of lesion area (either active or healing/healed) is also indicated on the plot. The samples (*n* = 4–5 biological replicates/condition/day) are shown divided by day post cohabitation (0 to 112 dpc). **(B)** Alpha diversity metrics of skin samples over time. The boxplots represent the diversity (Shannon’s index, **top**) or evenness (Pielou’s index, **bottom**) indexes of skin samples grouped (*n* = 4–5) by condition: control skin from the control condition (or SPF tank for 0 dpc), non-lesion or lesion area from the RMS cohabitation tank. Data are plotted as median with interquartile range

The main bacterial groups in the skin of SPF rainbow trout (0 dpc) were the genera *Stenotrophomonas*, *Chryseobacterium* and *Delftia*. This composition changed with time, with increasing community complexity. By 112 dpc, the skin microbiome included mainly *Collimonas* and *Delftia*, with lower abundances of *Arthrobacter*, *Chryseobacterium*, *Stenotrophomonas*, *Burkholderia* and *Paenarthrobacter* both in control and RMS cohabitant fish (Fig. 7A).

Importantly, at 50 dpc, cohabitants exhibited alterations in their skin microbiome. Active lesions showed a high prevalence of an ASV assigned to the “*Candidatus* Midichloria” genus. BLAST alignment revealed that the ASV had 100% identity to the known MLO 16S rRNA gene (GenBank: EU555284.1) (Suppl. Table 9). Lesion areas also had high abundances of an ASV assigned only at the class level as a gammaproteobacterium, and apparently healthy areas (non-lesion) displayed higher abundances of ASVs of this unclassified *g*ammaproteobacterium and “*Candidatus* Branchiomonas” as well. The “*Ca.* Branchiomonas” ASV matched with 100% identity with a previously reported 16S rRNA gene (GenBank: OQ930089.1) sequenced from the gill tissue of European whitefish (*Coregonus lavaretus*) [44]. The highest similarity assignment for the unclassified *g*ammaproteobacterium ASV also corresponded to a “*Ca.* Branchiomonas” sequence (GenBank: OQ930086.1) previously identified in the gills of Atlantic mackerel (*Scomber scombrus*) [44], although the identity score was relatively low (92.49%). Phylogenetic analyses confirmed that this unclassified gammaproteobacterium ASV was closely related to other members of the “*Ca*. Branchiomonas” genus (Suppl. Figure 6). Notably, the “*Ca.* Midichloria” ASV was not detected in fish from the control tank, whereas the two ASVs assigned to “*Ca.* Branchiomonas” were detected, albeit with low relative abundances (< 0.54% for “*Ca.* Branchiomonas” and < 0.03% for the uncl. gammaproteobacterium).

Consistent with relative abundance observations, the skin of cohabitants had lower values of diversity and evenness (Shannon, Pielou) compared to the control at dpc 50 (Fig. 7B), although the decrease was not statistically significant (Wilcoxon, *p* > 0.05). Except for the skin from affected fish towards the peak of infection (lesion and non-lesion sites at 50 dpc), a general increase in microbial community complexity was visible over time.

**Fig. 8:**
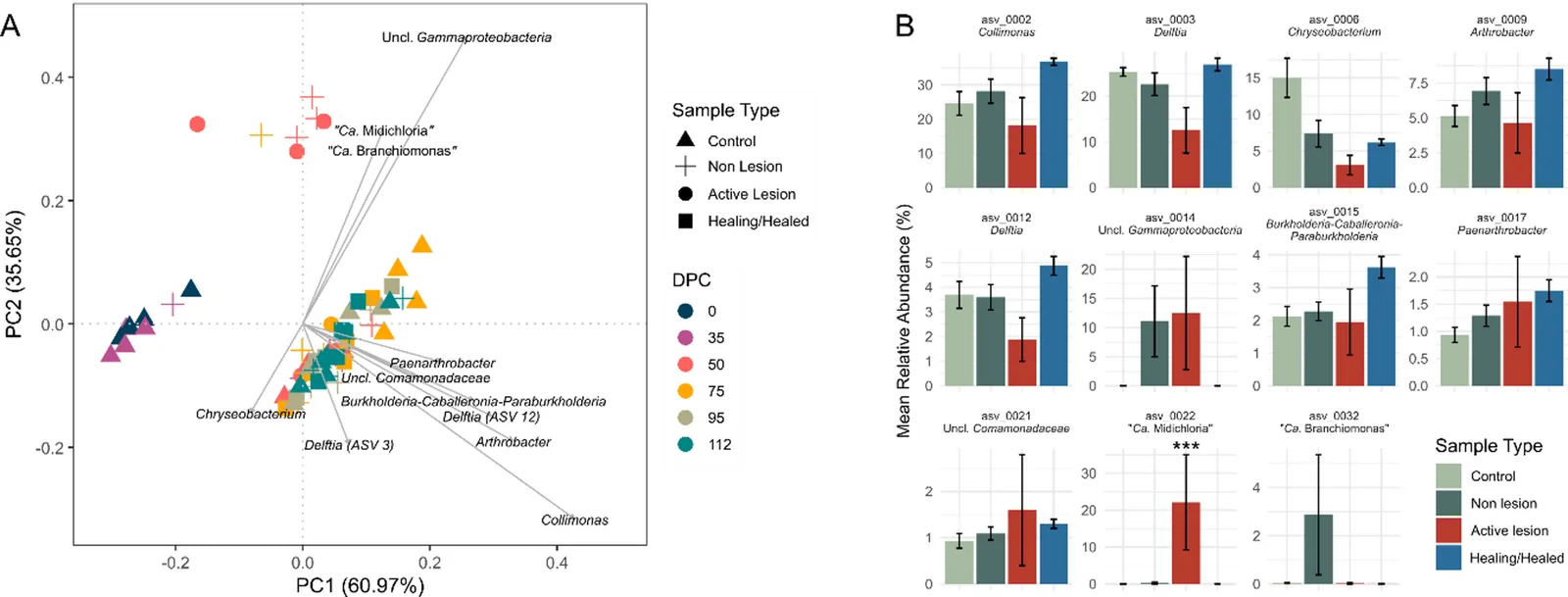
Beta diversity metrics of skin samples.** (A)** Robust Aitchison’s distance-based Principal Component Analysis (RPCA) ordination plot at the ASV level. Samples are coloured by sampling time point, and the condition group of the skin sample is differentiated by shape. Groups are either control (skin samples from the control condition, or SPF tank for 0 dpc), non-lesion skin areas from affected cohabitants, or lesion skin areas, which for analysis were further sub-grouped into active lesions or healing/healed lesions. The ASVs which are majorly contributing to the variability are also reported on the plot, with their taxonomic assignment to the lowest possible level and directionality indicators. **(B)** Relative abundance of ASVs majorly contributing to RPCA ordination variability in the skin microbial composition. The mean relative abundance (%) by group is reported as well as ASV taxonomic assignment to the lowest possible level. Skin samples categories are the ones used for the beta diversity analysis: control (either from SPF tank on dpc 0, or the control condition on the other timepoints), non-lesion, or lesion from affected RMS cohabitants, further subgrouped into active lesion or healing/healed lesion. Data are shown as mean relative abundance ± standard error (SE). “*Ca.* Midichloria” ASV was the only differentially abundant feature in active lesions (*** *p* ≤ 0.001) compared to control (ALDEx2, glm). No other ASV was highlighted as differentially abundant compared to the control (nor in non-lesion, active lesion, or healing/healed skin samples)

For the purpose of beta diversity analysis, we further categorized the lesion samples into active or healing lesions. Robust Aitchison distance PCA was used for ordination, with PC1 and 2 explaining 61.0% and 35.7%, respectively, of the total variation (Fig. 8A). Three distinct clusters were identified in this analysis. The majority of skin samples formed a single cluster, independently of time point, experimental condition, or skin sampling site, except for samples from 0 to 35 dpc coming mainly from control individuals that clustered separately along PC1. Interestingly, three lesion samples, together with a few non-lesion sites mainly from RMS cohabitants at 50 dpc, also formed a separate cluster. The defining features of this cluster were the ASVs assigned to “*Ca.* Midichloria”, unclassified gammaproteobacterium and “*Ca.* Branchiomonas”. Relative abundance plots by skin category showed that “*Ca.* Midichloria” ASV was almost exclusively present in active lesions, while the uncl. gammaproteobacterium and “*Ca.* Branchiomonas” ASVs were abundant in non-lesion skin samples (Fig. 8B).

### *“Ca.* Midichloria” is the only enriched ASV in active lesions, but dysbiosis is also present in apparently healthy areas

To investigate the differences between the microbiomes of the sampled skin areas, we performed differential abundance analysis of ASVs using ALDEx2. To this purpose, we once again sub-categorized lesion samples into active lesions and healing lesions based on the gross pathology of the sampled fish. This was done to discriminate between bacteria having a role in the development or in the healing phases of diseases, under the assumption that the pathogenic agent should not be present, or its abundance should decrease, during the healing phase, and opportunistic microbes might demonstrate an opposing trend. The generalized linear model (GLM) of ALDEx2 was used to include the time variable (dpc) as a covariate together with the skin sample type. The statistical test revealed that only one ASV was significantly enriched in active lesions compared to control skin, and it was the one assigned to “*Ca.* Midichloria” (*p* < 0.001) (Fig. 8B). Importantly, no statistically significant differences were present between the other skin sample types (non-lesions, healing/healed lesions) when compared to control samples.

Indeed, even though the ASVs of the unclassified gammaproteobacterium and “*Ca.* Branchiomonas” were found in elevated proportions in the skin of RMS-affected fish at peak pathology, they were not differentially abundant compared to the control. These ASVs were also found in the skin of control individuals (Suppl. Figure 7A), but their relative abundance only increased dramatically in RMS-affected cohabitants, around the time when the “*Ca.* Midichloria” ASV was predominant. Thus, to investigate the relationship between “*Ca.* Midichloria” and these ASVs, we performed a correlation analysis based on their relative abundances in each individual fish. In the case of the unclassified gammaproteobacterium (Suppl. Figure 7B), the relative abundance of the ASV seemed to be positively and significantly correlated with the relative abundance of MLO ASV in the same fish (Pearson’s *R* = 0.850, *p* < 0.001). This was not true for the “*Ca.* Branchiomonas” ASV (Pearson’s *R* = -0.017, *p* = 0.911), whose relative abundance increased in RMS-affected cohabitants, but not concurrently with that of “*Ca.* Midichloria”.

## Discussion

Despite decades of observation and study of this disease, the causative agent of red mark syndrome has eluded a definitive identification, likely largely because the putative pathogen could not be cultivated and isolated [6, 11, 12, 28]. In the present study we investigated the temporal dynamics of bacterial communities in water and skin of RMS-affected rainbow trout, collected from disease onset to healing during a semi-controlled experimental infection using individuals known to be pathogen free.

Previous culture-independent studies on RMS skin lesions, based on 16S rRNA gene sequencing, focused on the comparison of active lesions and control samples. The first report of this kind, based on 16S rRNA gene cloning and sequencing [11], identified a similar microbial composition between healthy and diseased skin areas, but with the MLO (then named RLO) highlighted as one of the few taxa present only in RMS lesions. A more recent study [21] found a significant enrichment of *Pseudomonas*, *Acinetobacter* and “*Candidatus* Midichloria” genera in RMS lesions. In contrast with these cross-sectional analyses, the present study has been designed to capture the temporal changes during development of disease. One earlier attempt of this experimental design had been briefly reported [53]. Here, we show that only lesions in the active stage have a different microbial composition compared to control skin, in contrast to pre-clinical samples, non-lesion areas, and healing lesions. These findings emphasize the importance of timing for sampling of RMS, and the need to examine active infections. Indeed, active lesions showed a reduced microbial diversity in favour of a few predominant ASVs. This approach revealed that only one ASV, assigned as “*Ca.* Midichloria” and aligning with 100% identity to the one identified by Lloyd et al. [11], was significantly enriched in actively developing RMS lesions. These results, corroborated by targeted molecular quantification over time, pinpoint the role of *Midichloria*-like organism (MLO) as a key driver of RMS in this study. Consistent with molecular postulates [23] and Hill’s framework [24], our temporal data link the specific enrichment of the MLO in active lesions, and its decline during healing, directly to the appearance, progression, and resolution of RMS. These observations add to the large body of empirical evidence that has been collected so far in the determination of the MLO as the causative agent of RMS.

Notably, the RMS-associated dysbiosis seemed to revert back to normality, with the skin microbiome of healing or healed lesions similar to control. This reversion to a control-like skin microbiome composition following RMS healing suggests that the rainbow trout skin microbiome may be capable of spontaneous restoration. However, it has been proposed that functional stability may be more relevant to ecological resilience and microbiome health than taxonomic similarity alone [54]. Future studies should therefore determine whether functional recovery parallels the observed taxonomic profile dynamics following RMS resolution.

Despite being more diverse than in active lesion skin, this control-like skin microbiome status comprised of relatively few genera. This may be due to our sampling method consisting of entire skin tissue sections, which was necessary to ensure the inclusion of intracellular microorganisms but that, compared to swabs, yields a higher proportion of host DNA, and may thus limit the observable ASV diversity when using a metabarcoding approach [55]. The overall low diversity of the skin microbiome in our experiment may also be due to the highly controlled nature of our experimental model, generated using SPF rainbow trout, compared to animals grown in environmentally more complex conditions [56].

An additional observation concerns the initial changes in the healthy microbiome of unaffected fish. The skin microbial composition of control fish at 35 dpc still closely resembled that of SPF fish at dpc 0, while RMS cohabitants showed early divergence, hinting at the possible role of the microbiome of RMS seeders in this change. However, towards the end of the trial, the microbiomes of asymptomatic skin from both control fish and cohabitants converged toward a similar, more diverse composition, independently from the exposure to seeder individuals. This could indicate that it is not only the more complex microbiome of seeder fish that modifies the skin bacterial composition, but likely this is also caused by other factors, such as the age of fish, or the transition to a different tank environment. Further supporting this, a reciprocal effect appeared to occur, as the introduction of rainbow trout to the experimental tanks was also associated with alterations in the original water microbiome composition.

An increase in mortality within a population has never been unequivocally associated with RMS [6]. Here, even though based on a relatively small number of samples, we observed a statistically significant reduction in survival probability (Kaplan-Meier) among RMS cohabitants compared to controls. As co-infections can occur in a non-pure cohabitation model [1], we tested organs of a proportion of dead individuals by bacterial culturing and by qPCR to determine whether other microorganisms might have contributed to mortality. Even though testing only a subsample of mortalities from the experiment for a relatively small panel of common pathogens constitutes a limitation of this study, our results nevertheless provided important insights into potential mechanisms associated with fish death. Two kidney samples of dead individuals were positive for MLO (Suppl. Table 4), and we detected high loads of “*Candidatus* Branchiomonas cysticola” in all gills, its known site of infection, but also in kidneys, where bacterial presence is not expected under healthy conditions, suggesting systemic infection. This was true not only in dead individuals, but also in the randomly collected rainbow trout during the experiment (50 dpc), where “*Ca.* Branchiomonas cysticola” was found in the kidney only in cases where the MLO was concomitantly detected in the same tissue (Suppl. Table 5). The detection of these bacteria in the internal organs is unexpected. Although extreme care was taken to aseptically dissect tissues, the possibility of contamination from adjacent tissues and tank environment cannot be entirely excluded; however, these observations warrant further investigation into the capability for systemic dissemination of the MLO and “*Ca*. Branchiomonas cysticola”, particularly in the context of co-infections.

In agreement with this, increased (but non-significant) relative abundances of an uncl. gammaproteobacterium and “*Ca.* Branchiomonas”-assigned ASVs were a prominent feature of the microbial dysbiosis observed in seemingly healthy skin areas (non-lesions) of infected fish. Notably, the uncl. gammaproteobacterium ASV was also closely related to “*Ca*. Branchiomonas” sequences. The genus “*Ca*. Branchiomonas” is, according to the GTDB taxonomic revision, within the *Gammaproteobacteria* class [57]. Moreover, a “*Ca.* Branchiomonas” species, “*Ca.* B. cysticola”, which we detected by qPCR in the gills and kidneys of dead individuals in this study, is a putative pathogen which was linked to epitheliocystis in Atlantic salmon (*Salmo salar*) [30, 58–60]. “*Ca*. Branchiomonas” is known to be part of the common gill microbiome in Atlantic salmon [60–62] and other non-salmonid fish species [63, 64], but is not commonly reported as part of the salmonid skin microbiome. In rainbow trout, “*Ca.* Branchiomonas” has been found in the gills of healthy or asymptomatic individuals [65, 66], and in both healthy and clinically affected fish in complex, multifactorial disease cases [67]. Given that the bacterium is found as part of the healthy gill microbiome of several teleost species as well as associated with the insurgence of disease, it is currently unclear whether “*Ca*. Branchiomonas” species are primary pathogens, or if they have a role in complex infectious diseases, or if they act as opportunistic organisms able to proliferate in hosts affected by other disease processes. The fact that we were able to detect “*Ca.* Branchiomonas” not only in skin and gills, but also, atypically, in internal organs of fish that succumbed during the trial (Suppl. Table 4) and RMS-affected fish (Suppl. Table 5), seems to support that this bacterium may be a facultative or opportunistic pathogen causing disease in rainbow trout in vulnerable conditions. Interestingly, our detection of “*Ca*. Branchiomonas” in RMS skin lesions and in the kidneys of RMS-affected rainbow trout is consistent with observations in a similar condition, red skin disease (RSD) in Atlantic salmon, where “*Ca.* Branchiomonas cysticola” was likewise detected not only in skin ulcers, but also in internal organs of affected fish [31].

Despite their increased abundance in some of the non-lesion and lesion areas, these two ASVs were not statistically differentially abundant in any skin sample type from RMS cohabitants compared to controls. Indeed, although in low abundances, the uncl. gammaproteobacterium and the “*Ca.* Branchiomonas” ASVs were both detected in control fish during the experiment (Suppl. Figure 7A). Regardless of whether they were already present on SPF fish, or if they were acquired later during the experimental trial, this suggests that they can coexist with their host as part of a healthy skin microbiome, but it was only in the skin of RMS cohabitants that they were observed to proliferate. In particular, the increase in relative abundance of the uncl. gammaproteobacterium was significantly correlated with that of the “*Ca.* Midichloria” (MLO), possibly suggesting a synergistic relationship. The observation that potentially pathogenic taxa, such as “*Ca.* Branchiomonas” species, were detectable at low abundance even in healthy control fish, yet proliferated only in RMS cohabitants, is consistent with the pathobiome model [68]. The healthy fish skin mucus harbours a resident commensal community involved in the maintenance of the host’s health status through niche competition, production of antagonistic compounds, and stimulation of immune responses [69, 70]. The disruption of this homeostasis, whether by primary infection, stress, or tissue damage, can reduce resistance to colonisation, enabling low-abundance opportunistic microorganisms to exploit the altered microenvironment and proliferate, contributing to the resulting dysbiosis [71, 72].

In our experimental cohabitation trial, RMS development followed the expected course, with a latency from the start of cohabitation [1, 15] before the appearance of early-stage lesions around 50 dpc, followed by peak pathology between 50 and 75 dpc, and finally a gradual recovery with full healing by day 112. Observations of gross pathology were corroborated using histopathology, which revealed that lesions conformed to RMS disease characteristics [73, 74], including severe lymphohistiocytic infiltration into all dermal layers, scale resorption, oedema and congestion of blood vessels (Fig. 3D).

For a holistic investigation of the involvement of MLO in RMS, we also investigated presence of MLO in skin through a targeted strategy in addition to the 16S rRNA gene sequencing approach, by quantifying the bacterium in skin via qPCR. Coherently with previous research [15], MLO was detected in the skin of RMS-affected fish, mainly in lesions and, to a lesser extent, in non-affected skin areas of infected fish, but not in control fish (Fig. 4). Interestingly, we observed the highest quantity of MLO at 50 dpc, the sampling timepoint immediately preceding the peak of disease. This apparent relationship between the load of MLO and the presence of skin lesions pathology strongly support the current hypothesis that this bacterium has a central role in the development of RMS lesions in rainbow trout. Accordingly, when the lesions started to heal, the load of MLO decreased until becoming undetectable at the time when the fish were healing or completely healed, at 112 dpc.

Targeted qPCR also elucidated the temporal dynamics of MLO in water during the entire course of infection and RMS development. The pattern of MLO load in the water suggests two distinct waves of MLO shedding, aligning with the hypothesis of disease transmission from seeders to cohabitants through water. Accordingly, the first peak (20 dpc) likely reflected the shedding from RMS-affected seeder fish introduced at 0 dpc, which were already showing active lesions and may have released MLO into the water approximately 20 days later. Then, after a latency period during which the lesions of seeders began to heal, all cohabitants developed early signs of RMS before the sampling on 50 dpc, potentially leading to the second major peak (75 dpc) of MLO in water. The consistent interval from active lesions to shedding further supports the hypothesis that the MLO might be the causative agent of the disease. Previous efforts to detect the MLO through eDNA yielded sporadic low-level detections and mainly during the late stages of infection [22, 29]. This represents the first report of successful molecular quantification of the MLO in the rearing water of RMS-affected fish, detected using eDNA along the entire duration of the experimental trial, and provides interesting insight into how this microbe might be transmitted between cohabitants, given its characterization as an intra-cellular microbe. However, as qPCR-based quantification can be influenced by factors unrelated to the experimental variables of interest [75, 76], future independent studies reproducing these results, both in rainbow trout skin and in water, would further strengthen and validate this evidence.

To complement the targeted detection of MLO, we performed 16S rRNA gene amplicon sequencing on the same water samples to characterize broader microbial community dynamics throughout the course of the experiment. Microbial populations in water seemed to be shaped more by time than by the disease or non-disease condition. Several of the main components of the water microbiome, namely *Flavobacterium*, *Undibacterium*, *Rhodoferax*, *Hydrogenophaga* and *Flectobacillus* genera, had previously been reported as abundantly present in rainbow trout aquaculture water, both in tanks where RMS was present or absent [22]. Concerning the putative pathogen detection, we showed that 16S rRNA metabarcoding, at least at the sequencing depth at which we tested it, was unable to detect the ASV of MLO in water, in contrast to the targeted qPCR analysis. A similar phenomenon has been previously reported by Bruno et al. [22]. This is most likely due to the much higher prevalence of other bacteria in the water, and ‘outcompeting’ of the genetic material from these taxa for 16S sequencing, despite the appearance of appropriate sequencing depth suggested by rarefaction curves. The same reduced sensitivity of the 16S rRNA analysis compared to qPCR for pathogen detection in water has also been observed in experimental trials with another intracellular bacterium, *Renibacterium salmoninarum*, even under high bacterial loads (Villamil-Alonso & Cuenca, personal communication).

This study is not without limitations. In particular, the analysis of 16S rRNA gene amplicons can be limited by primer bias which might lead to the underrepresentation of key bacterial taxa, potentially explaining, for instance, why the MLO was not detected in an early study using this method [1]. Although universal primers have notably improved over time, the risk of missing relevant bacteria still exists and, for this reason, untargeted methods such as metagenomic sequencing should be prioritized in future studies. Similarly, the lack of completeness of reference libraries limits taxonomic classification of identified ASVs. While a metagenomic approach would still be limited by reference materials, it would allow not only a more comprehensive description of the skin microbiome but could potentially expand our current knowledge on the genome of the putative pathogen, MLO. Understanding more about its genomic content would prove crucial in the development of more specific diagnostic assays, in situ probes, and even preventive and therapeutic strategies.

In conclusion, this study corroborates and significantly extends previous attempts at characterization of the bacterial composition of RMS lesions. We provide strong evidence supporting the role of the *Midichloria*-like organism in the development of RMS skin lesions in rainbow trout, with a perspective on the entire disease course. By combining molecular approaches such as 16S rRNA profiling and qPCR, we describe disease progression and the associated microbial shifts, and show that MLO abundance correlates with lesion severity and declines upon healing. No other bacteria correlate significantly with the progression of the pathology. Moreover, the detection of MLO in water further supports the supposed role of this bacterium in transmission. At the same time, the increased mortality, dysbiosis, and co-occurrence with opportunistic taxa suggest that RMS may predispose fish to secondary infections, potentially triggering more complex, multifactorial disease outcomes. Our findings reinforce the central role of the MLO in RMS pathogenesis, and highlight the need for further genomic and functional studies.

## Supplementary Information

Below is the link to the electronic supplementary material.

Supplementary Material 1

Supplementary Material 2

## Data Availability

The data supporting the findings in this study are provided in the Supplementary Tables. The raw sequencing data have been deposited in the Sequence Read Archive (SRA), BioProject PRJNA1354637 (BioSamples SAMN52950823-SAMN52950956). The scripts used to process the data are available on GitHub: https://github.com/zrngiulia/Red-mark-syndrome-16S.
