# Supplementary material for: From onset to healing: temporal dynamics of microbial communities pinpoint Midichloria-like organism’s key role in the development of the fish skin disease red mark syndrome
Source: Anim Microbiome. 2026 Aug 19;8:95. doi: 10.1186/s42523-026-00588-z (PMC13488072; doi:10.1186/s42523-026-00588-z)
Supplement: Supplementary file 1 [file 42523_2026_588_MOESM1_ESM.pdf]

Supplementary figures

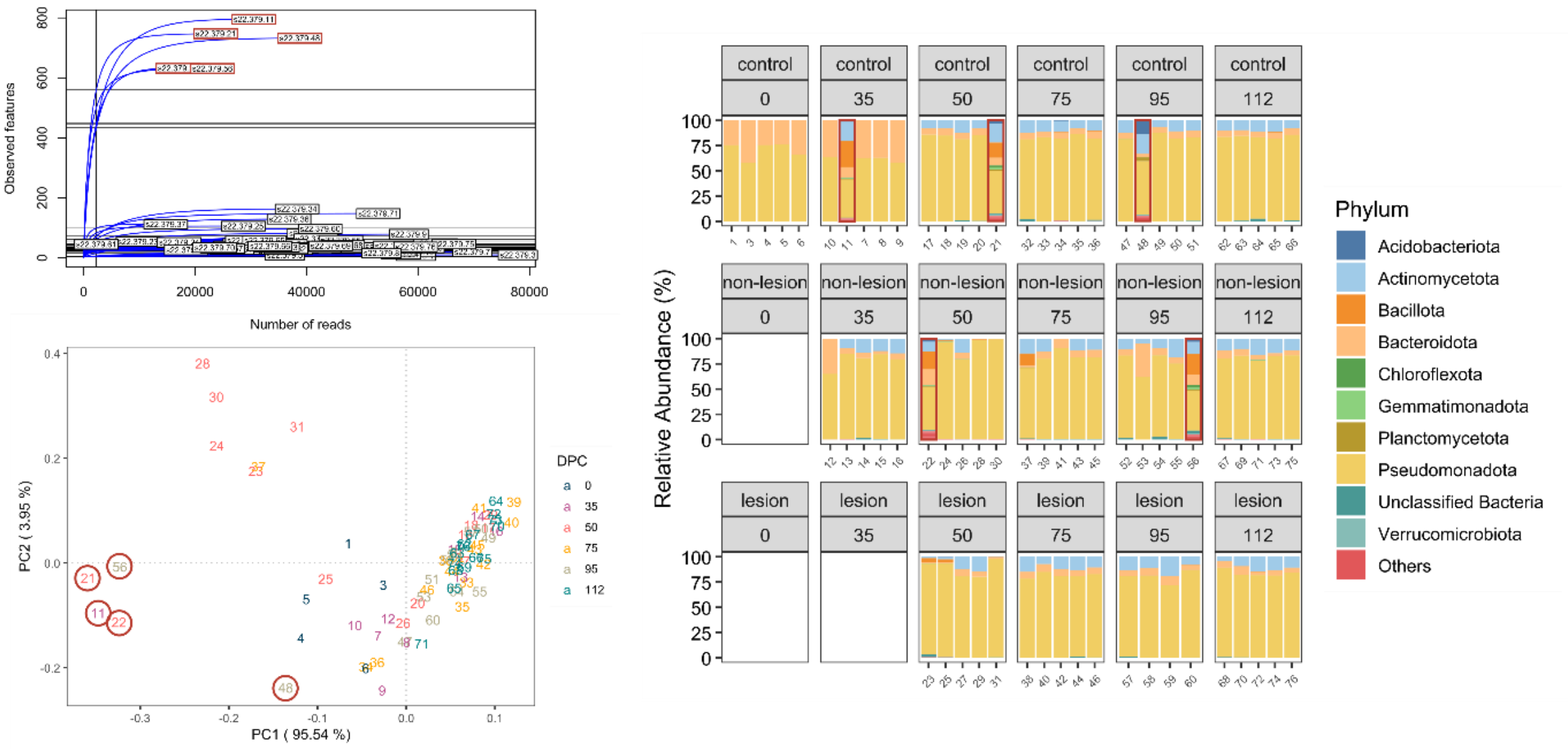

Suppl. Figure 1. Five outlier samples were discarded from analysis (one control from 35 dpc, one control from 50 dpc, one non-lesion from 50 dpc, one control from 95 dpc, one non-lesion from 95 dpc).They were excluded based on: the number of observed ASVs, between 600 and 800, while the rest of skin samples were all below 200 ASVs (rarefaction curves, **top left**); outlier appearance when observing relative abundances at phylum level (stacked barplots, **right**); clustering separately from other samples, their separation on PC1 explaining most of the observed variation (95.54%), and causing all other samples to collapse closer together, thus impairing meaningful interpretation of results (Robust Aitchison's distance-based PCA plot, **bottom left**).

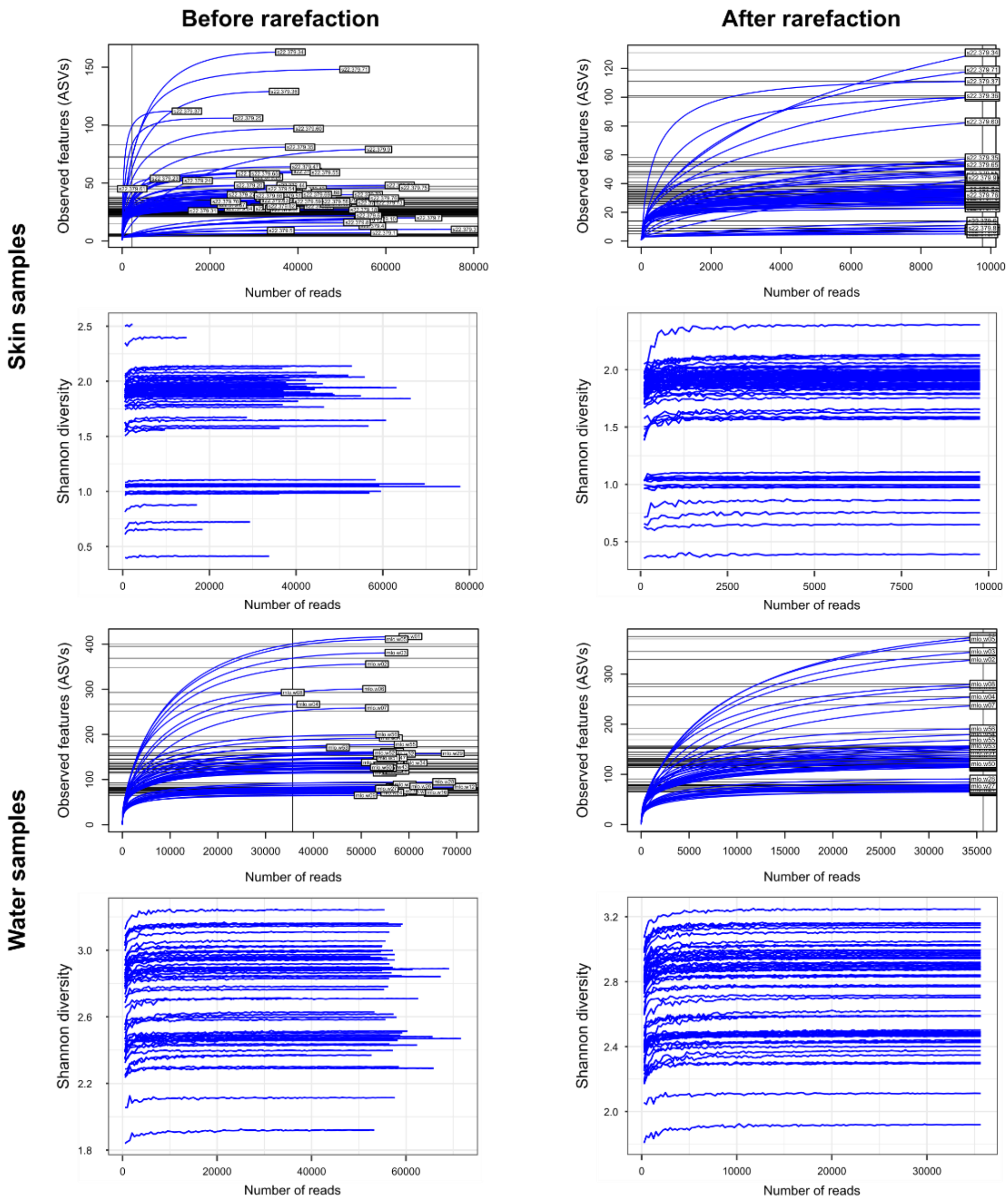

Suppl. Figure 2. **Rarefaction curves before and after rarefaction of the skin and water samples datasets.** Rarefaction curves are based on observed ASVs or Shannon diversities. Curves are shown before **(left)** or after **(right)** rarefaction, at 9756 reads/sample and 35658 reads/sample for skin and water samples, respectively. Note that for skin, after rarefaction, one sample with an insufficient number of reads (sample s22.379.61, 2278 reads) was discarded.

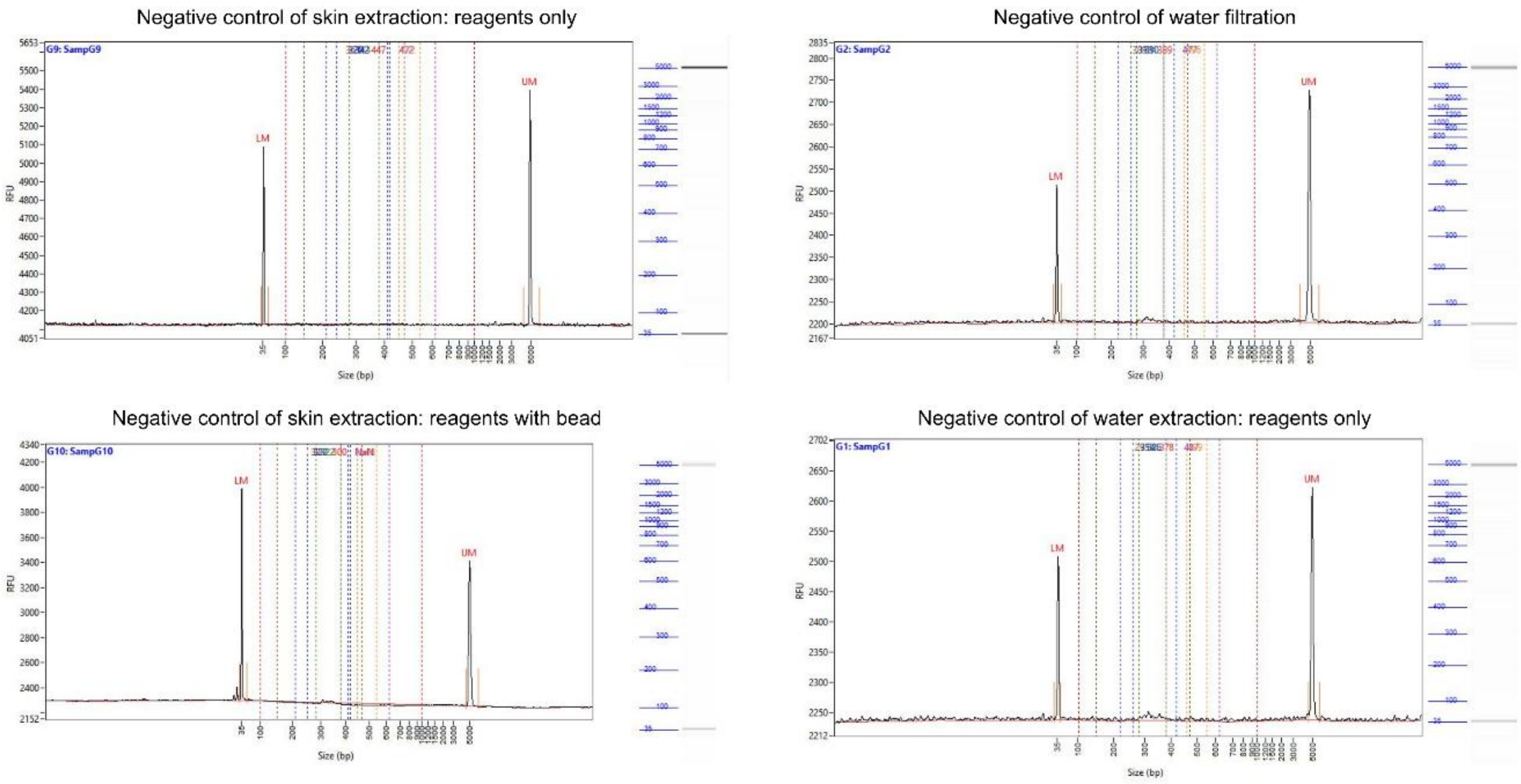

Suppl. Figure 3. **16S rRNA gene PCR amplification for library preparation failed to yield amplicons in all negative control samples.** After 16S rRNA gene PCR, the resulting amplification products were subjected to quality control using a capillary electrophoresis system (Agilent 5400). Results of PCR amplification for two negative controls of skin extraction, containing either reagents only (MagMAX Microbiome Ultra Nucleic Acid Isolation Kit, Applied Biosystems) **(top left)** or reagents with the addition of the 0.5 cm sterile stainless-steel bead (Qiagen) **(bottom left)** are shown. Amplicons were also absent from the negative controls of extraction for the water samples. Negative control of extraction from an unused 0.22 µm PVDF filter membrane **(top right)** or reagents only **(bottom right)** did not yield an amplicon suitable for sequencing. Since the PCR failed to yield an adequate amplification product, the negative controls were not carried forward to sequencing.

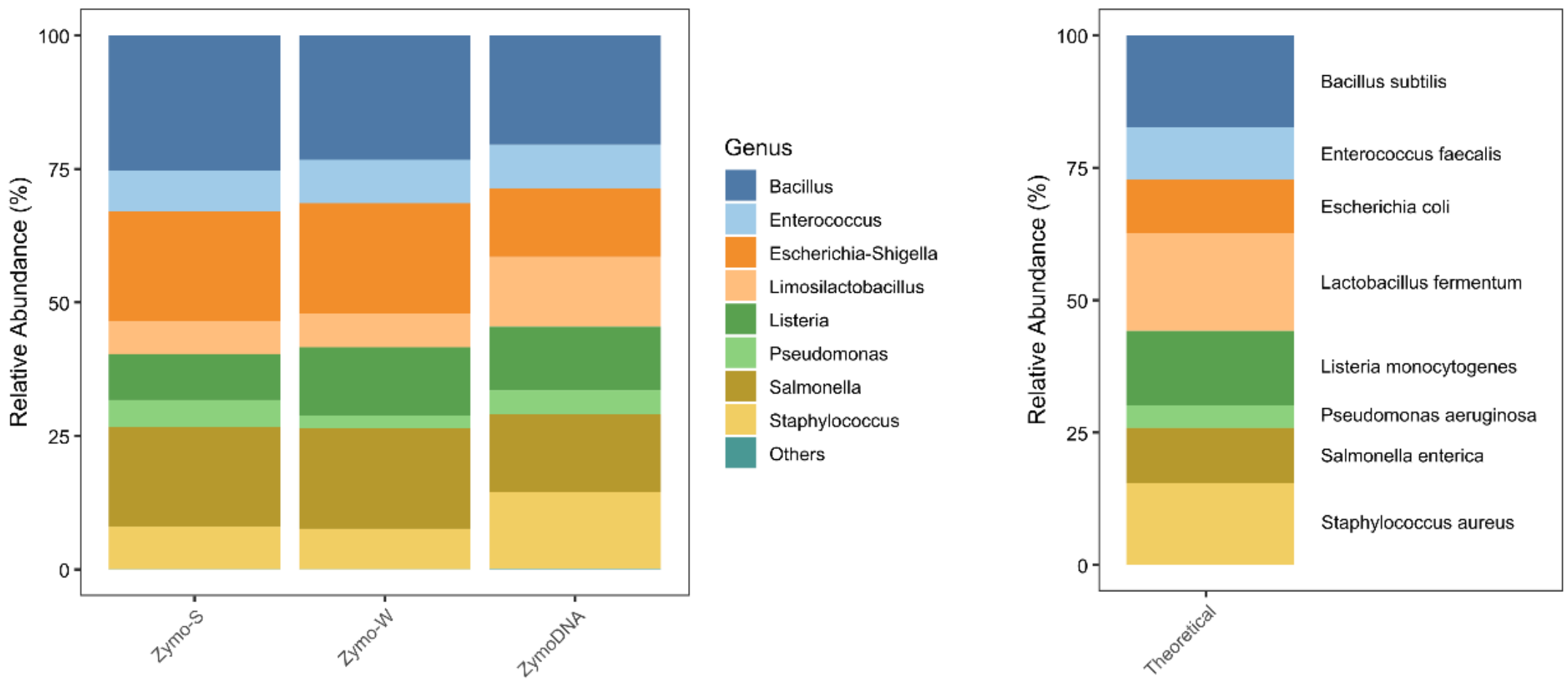

Suppl. Figure 4. **Microbial composition (%) of mock community standards.** Relative abundances (%) are based on raw counts (non-rarefied datasets). **Zymo-S**: ZymoBIOMICS™ Microbial Community Standard (#D6300, Zymo Research) aliquot extracted with skin samples. **Zymo-W**: ZymoBIOMICS™ Microbial Community Standard (#D6300, Zymo Research) aliquot extracted with water samples. **ZymoDNA**: ZymoBIOMICS™ Microbial Community DNA Standard (#D6305). On the right, the theoretical composition (%) of 16S rRNA gene provided by the manufacturer.

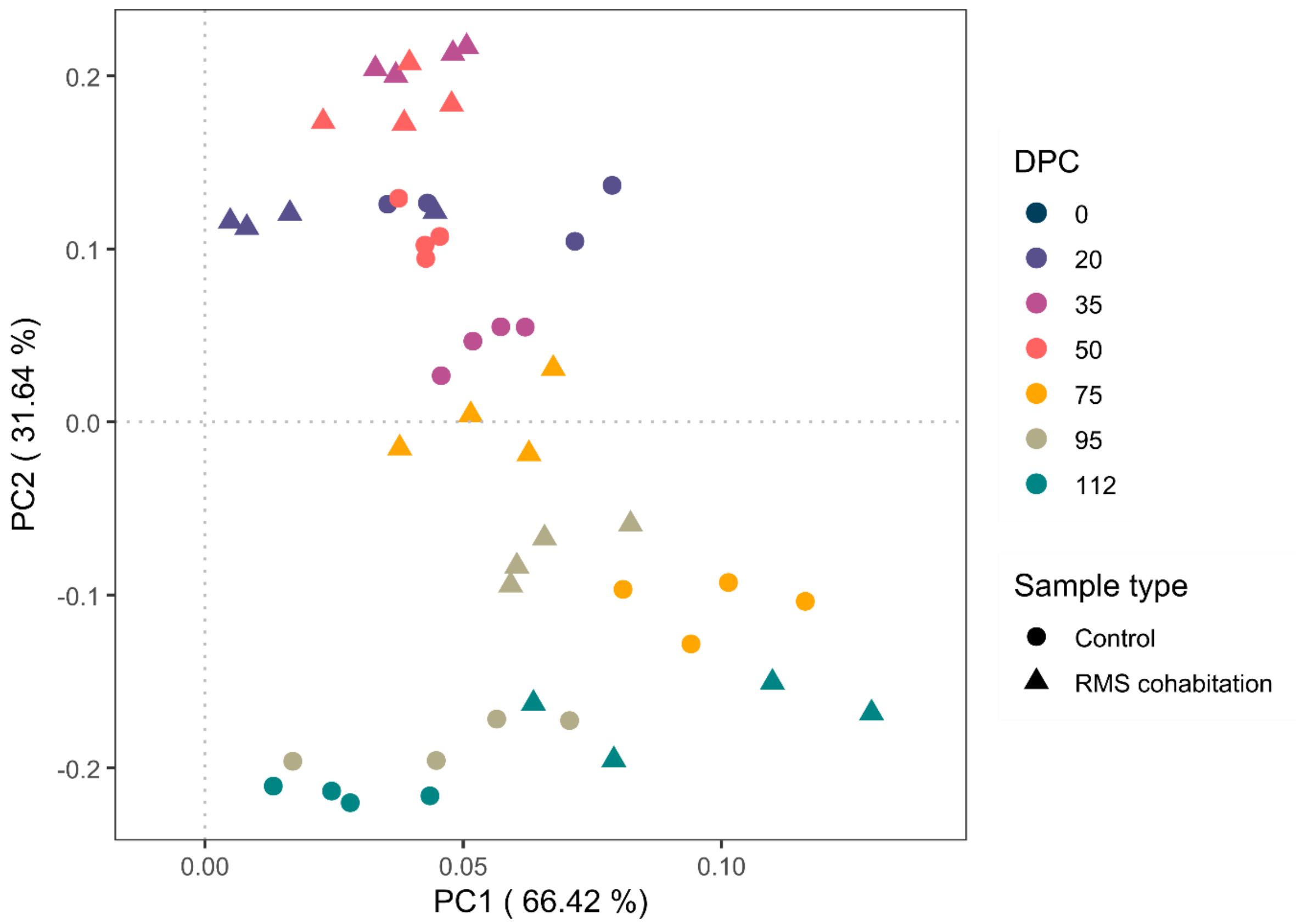

Suppl. Figure 5. Zoomed-in view of the beta diversity metrics of water samples. Robust Aitchison's distance-based Principal Component Analysis (RPCA) ordination plot, ASV-level analysis. Technical replicates of water filtration (*n* = 4) are plotted as separate data points. Samples are coloured by sampling time point, and the condition (control or RMS cohabitation) is differentiated by shape. The original plot (Figure 6B) was zoomed in (0 < PC1 < 0.14) to exclude datapoints corresponding to 0 dpc, thereby facilitating visualization of the relative positioning of datapoints from later time points (20–112 dpc) according to time and tank of origin.

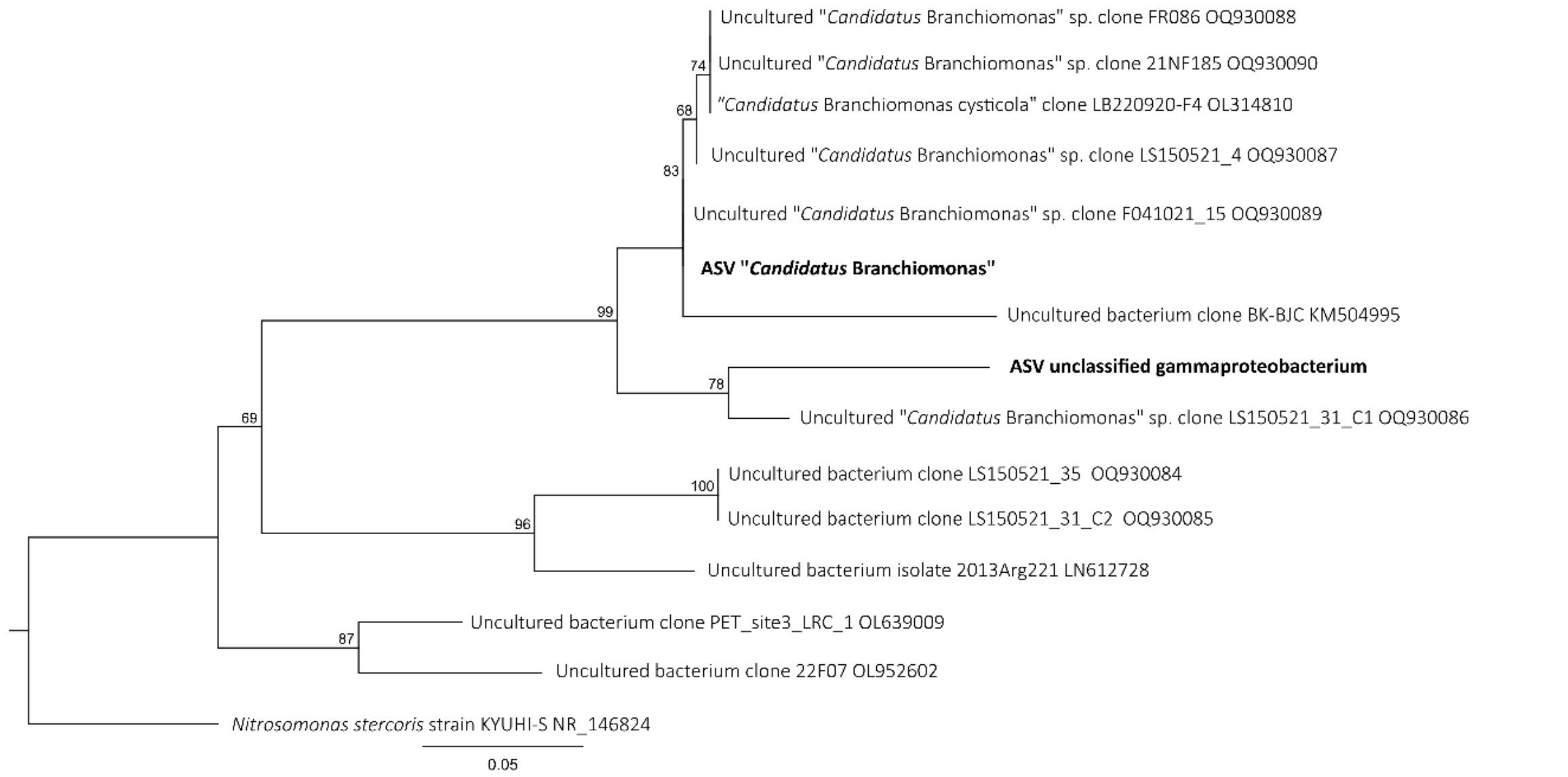

Suppl. Figure 6. Maximum likelihood (ML) phylogenetic tree of selected ASVs from the present study and *Branchiomonaceae,* based on partial 16S rRNA gene sequences. Selected sequences from Mjølnerød et al., 2024 were included (*n* = 12), as well as the outgroup *Nitrosomonas stercoris* (NR_146824), to determine the phylogenetic placement of the "*Ca*. Branchiomonas" ASV and the unclassified gammaproteobacterium ASV from the present study. Bootstrap values calculated from 100 replicates are reported on the nodes. The phylogenetic analysis confirms that the unclassified gammaproteobacterium ASV clusters in the "*Ca*. Branchiomonas" clade.

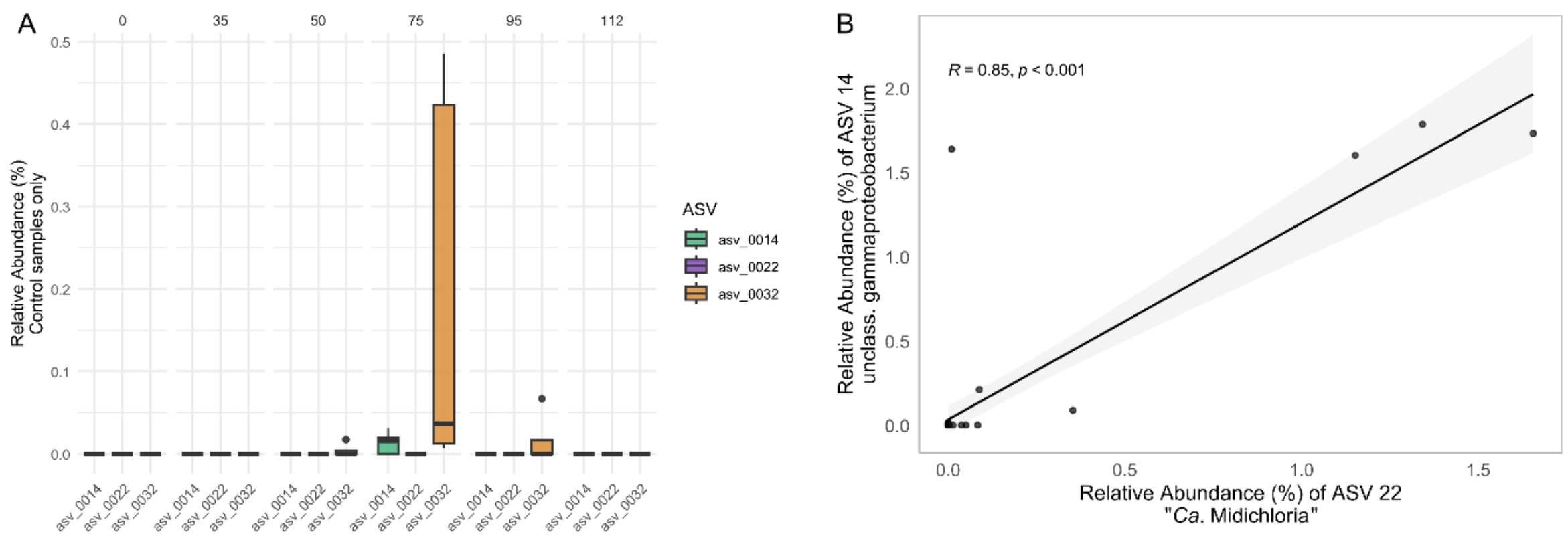

Suppl. Figure 7. Despite being present in also in control samples, the relative abundance of unclassified gammaproteobacterium (ASV 14) and "*Ca.* Branchiomonas" (ASV 32) abundance only increased in cohabitants, where "*Ca.* Midichloria" (ASV 22) is present. A. Boxplots show the relative abundance (%), calculated from raw counts (not rarefied) of ASVs corresponding to the unclassified gammaproteobacterium (ASV 14), "*Ca*. Midichloria" (ASV 22) and "*Ca*. Branchiomonas" (ASV 32) in control samples only (SPF fish on 0 dpc; fish from the control condition on 35 to 112 dpc). Data are plotted as median with interquartile range. **B.** Relative abundances (%) calculated from raw counts (not rarefied) of ASVs corresponding to "*Ca*. Midichloria" (ASV 22) and unclassified gammaproteobacterium (ASV 14), averaged by individual (i.e., non-lesion and lesion from the same fish), following a linear fit in the dotplot. Linear regression and data visualization were performed after logarithmic transformation (log10).
